# Fast event-based electron counting for small-mol­ecule structure determination by MicroED

**DOI:** 10.1107/S2053229624012300

**Published:** 2025-02-21

**Authors:** Niko Vlahakis, Songrong Qu, Logan S. Richards, Lygia Silva de Moraes, Duilio Cascio, Hosea M. Nelson, Jose A. Rodriguez

**Affiliations:** a Department of Chemistry and Biochemistry UCLA-DOE Institute for Genomics and Proteomics STROBE NSF Science and Technology Center University of California Los Angeles (UCLA) Los Angeles CA 90095 USA; b Division of Chemistry and Chemical Engineering California Institute of Technology,Pasadena CA 91125 USA; University of Delaware, USA

**Keywords:** crystal structure, electron counting, MicroED, EBEC, electron dif­frac­tion

## Abstract

Electron counting helped realize the resolution revolution in single-particle cryoEM and is now accelerating the determination of MicroED structures. Its advantages are best demonstrated by new direct electron detectors capable of fast (kilohertz) event-based electron counting (EBEC). This strategy minimizes the inaccuracies introduced by coincidence loss (CL) and promises rapid determination of accurate structures. We used the Direct Electron Apollo camera to leverage EBEC technology for MicroED data collection. Given its ability to count single electrons, the Apollo collects high-quality MicroED data from organic small-mol­ecule crystals illuminated with incident electron beam flux densities as low as 0.01–0.045 e^−^/Å^2^/s. Under even the lowest flux density (0.01 e^−^/Å^2^/s) condition, fast EBEC data produced *ab initio* structures of a salen ligand (268 Da) and biotin (244 Da). Each structure was determined from a 100° wedge of data collected from a single crystal in as few as 50 s, with a delivered fluence of only ∼0.5 e^−^/Å^2^. Fast EBEC data collected with a fluence of 2.25 or 3.33 e^−^/Å^2^ also facilitated a 1.5 Å structure of thio­strepton (1665 Da). While refinement of these structures appeared unaffected by CL, a CL adjustment applied to EBEC data further improved the distribution of intensities measured from the salen ligand and biotin crystals. However, CL adjustment only mar­ginally improved the refinement of their corresponding structures, signaling the already high counting accuracy of detectors with counting rates in the kilohertz range. Overall, by delivering low-dose structure-worthy data, fast EBEC collection strategies open new possibilities for high-throughput MicroED.

## Abstract 2

Fast readout event-based electron counting (EBEC) is a promising detection strategy to determine accurate *ab initio* structures of beam-sensitive small mol­ecules by MicroED. A fast EBEC approach enhances the dynamic range of MicroED data by limiting the likelihood of coincidence loss (CL) – the undercounting of electrons due to their spatially and temporally unresolved arrival on a direct electron detector. As implemented by new counting detectors, fast EBEC allows structure-worthy data sets to be captured from individual small-mol­ecule crystals in under a minute.

## Introduction

Microcrystal electron dif­frac­tion (MicroED), also referred to as three-dimensional electron dif­frac­tion, is sought out for its ability to inter­rogate a variety of micro- or nanoscale crystallites (Saha *et al.*, 2022 ▸), including those of proteins (Shi *et al.*, 2013 ▸), peptides (Rodriguez *et al.*, 2015 ▸), small mol­ecules (Jones *et al.*, 2018 ▸), and materials (Wang *et al.*, 2018 ▸). One key to the success of MicroED has been its adoption of existing crystallographic approaches and cryoEM instrumentation for the determination of atomic structures (Rodriguez *et al.*, 2017 ▸). Over the past decade, the typical MicroED experiment has relied on the use of a transmission electron microscope (TEM) equipped with an electron source operating at 200–300 keV and fitted with a scintillator-based pixelated detector. In this way, TEMs have been, without much alteration, readily adaptable to MicroED experiments at room tem­per­a­ture or under cryogenic conditions (Saha *et al.*, 2022 ▸). MicroED has also benefitted from the robustness of crystallographic theory and its application, broadly adopting the implementation of crystallographic data reduction and refinement software (Kabsch, 2010 ▸; Winter *et al.*, 2018 ▸; Dolomanov *et al.*, 2009 ▸; Adams *et al.*, 2010 ▸; Murshudov *et al.*, 2011 ▸; Emsley & Cowtan, 2004 ▸; Sheldrick, 2008 ▸, 2015*a* ▸,*b* ▸).

Several recent advances have expanded the applicability of MicroED. These include updated sample preparation methodologies, such as focused ion beam milling (Duyvesteyn *et al.*, 2018 ▸; Martynowycz *et al.*, 2019 ▸; Parkhurst *et al.*, 2023 ▸), pressure-assisted sample deposition and the use of microarray robotics (Zhao *et al.*, 2021 ▸; Delgadillo *et al.*, 2024 ▸). Likewise, software developments have improved the likelihood of determining challenging beam-sensitive samples and, in some cases, informing on their chiral nature (Saha *et al.*, 2022 ▸; Palatinus *et al.*, 2017 ▸; Brázda *et al.*, 2019 ▸), com­plementing electron nanobeam and serial dif­frac­tion approaches that improve throughput, and extract crystallographic information from minuscule collections of mol­ecules (Bücker *et al.*, 2020 ▸; Gallagher-Jones *et al.*, 2020 ▸; Hogan-Lamarre *et al.*, 2024 ▸). Some of these new developments have specifically leveraged new commercial or experimental (Saha *et al.*, 2023 ▸) direct electron detectors (DEDs), illuminating the promise of these types of sensors for dif­frac­tion.

The growing adoption of DEDs for dif­frac­tion data collection echoes their successful application to single-particle cryoEM (Wu *et al.*, 2016 ▸). However, dif­frac­tion experiments represent a unique challenge for DEDs, particularly when performing electron counting, because of coincidence loss (CL) (Li *et al.*, 2013 ▸; McMullan *et al.*, 2014 ▸; Gallagher-Jones *et al.*, 2019 ▸; Hattne *et al.*, 2023 ▸). When imaging in real space, a DED can anti­cipate a nearly flat illumination across its surface. In this mode, it must count individual events landing at a given rate, with relatively equal likelihood, on any one of its pixels (Li *et al.*, 2013 ▸). This allows inter­pretable images to be recorded at low flux, with a limited dynamic range detector. By contrast, dif­frac­tion from highly ordered crystals requires accurate counting across a wide dynamic range. A large number of electrons must be accurately counted at high-intensity reflections, while single electrons must be registered at the weakest reflections.

Since the distribution of crystal reflection intensities on a detector is difficult to anti­cipate *a priori*, any pixel on the sensor must be able to deliver the full dynamic range at any given time. This presents a challenge for most commercially available DEDs, which have low readout and inter­nal counting or frame rates that fundamentally limit the number of electrons measurable per pixel per second. Exceeding these limits results in CL, which effectively narrows the dynamic range of observable signal. Despite these challenges, several studies have demonstrated the utility of electron counting for electron dif­frac­tion data collection. For example, in 2019, a K2 detector was used to chart the nanoscale mosaicity of peptide crystals using nanobeam dif­frac­tion (Gallagher-Jones *et al.*, 2019 ▸); challenges with CL were notable under those conditions. Follow-up work in 2020 led to the structure of a hexa­peptide nanocrystal determined *ab initio* from electron nanobeam dif­frac­tion data, also collected on a K2 detector in counting mode (Gallagher-Jones *et al.*, 2020 ▸). More recently, similar detectors have been used to determine *ab initio* structures of macromolecules (Martynowycz *et al.*, 2022 ▸), with specific data collection settings implemented to minimize CL while retaining high-resolution signal (Hattne *et al.*, 2023 ▸; Clabbers *et al.*, 2022*b* ▸).

Most MicroED experiments using DEDs in counting mode have required severe restriction of incident electron beam flux on the crystal. This lowering of beam flux is often com­pen­sated for by extending integration and overall data collection times (Martynowycz *et al.*, 2022 ▸; Clabbers *et al.*, 2022*a* ▸,*b* ▸; Takaba *et al.*, 2021 ▸). The need for drastic flux reduction depends on the type of DED used, and its inter­nal count rate. For example, new hybrid pixel detector (HPD) technologies have overcome these limitations to enable fast serial electron dif­frac­tion (SerialED) data collection (Bücker *et al.*, 2020 ▸). However, the advantage afforded by the speed and sensitivity of HPDs is offset by their larger (often in the range of 50–80 µm^2^) pixel size and limited sensor pixel density com­pared to monolithic active pixel sensors. In detectors such as the Apollo (Direct Electron), on-chip event-based thresholding and registration addresses the need for high-speed counting while reducing the burden of handling and processing large amounts of raw data (Peng *et al.*, 2023 ▸). This implementation permits kilohertz-rate event-based electron counting (EBEC). The use of field programmable gate arrays (FPGA) for down­stream centroid detection further enables on-the-fly super-resolved event localization (Peng *et al.*, 2023 ▸).

Here we explore the advantages of leveraging fast EBEC technology, as implemented by the Apollo detector (Peng *et al.*, 2023 ▸), for small-mol­ecule MicroED. We focus on small-mol­ecule crystals since they represent a general challenge for electron counting procedures by producing fewer more intense reflections than their macromolecular counterparts. We analyze a fluence regime that can enable high-throughput data collection and the rapid determination of accurate atomic structures from beam-sensitive crystals in semi-automated fashion.

## Materials and methods

### Samples and sample preparation

Crystal samples were obtained and prepared for MicroED analysis as follows:

(1) *Salen ligand*: (*S*,*S*)-Jacobsen’s salen ligand was pur­chased from Sigma. The powder was dissolved in a solution com­posed of di­chloro­methane and ethanol (1:2 *v*/*v*), and the solution was left to evaporate slowly. After approximately 1 d, following drying of the solvent, thin rod-shaped crystals of a pale-yellow color were observed. The population of crystals varied in size, including some of suitable size (∼0.5 mm long) for X-ray dif­frac­tion, but many much smaller. TEM grids were briefly dusted with crystalline powder, producing a distribution of submicron-thick crystals suitable for MicroED. The anti­cipated salen ligand crystal polymorph was in the space group *P*2_1_2_1_2_1_ (CCDC ID: 129337), with unit-cell constants *a* = 6.78, *b* = 18.33, *c* = 27.75 Å, α = 90, β = 90 and γ = 90° (Yoon *et al.*, 1997 ▸).

(2) *Biotin*: biotin was purchased as a powder from Sigma. A saturated solution was prepared in water heated to 100 °C. The solution was allowed to return slowly to ambient tem­per­a­ture, during which time colorless needle-shaped crystals formed. The crystal suspension was diluted tenfold in ethanol and saved for subsequent TEM sample preparation. Grids were prepared by pipetting 2 µl directly from this suspension, allowing the sample to settle on the grid for approximately 30 s, and then wicking off excess solvent with filter paper, leaving submicron thin needle-shaped crystals on the grid for MicroED analysis. The anti­cipated biotin crystal polymorph was in the space group *P*2_1_2_1_2_1_ (CCDC ID: 1111310), with unit-cell constants *a* = 5.24, *b* = 10.35, *c* = 21.04, α = 90, β = 90 and γ = 90° (DeTitta *et al.*, 1976 ▸).

(3) *Thio­strepton*: 30 mg of commercially acquired thio­strepton was dissolved in 1.95 ml of a 24:1 (*v*/*v*) chloro­form–isoamyl alcohol solution. 390 µl of ethanol and 195 µl of 100% glycerol were added and mixed into the solution. The solution was allowed to evaporate slowly at ambient tem­per­a­ture, and after 4 d, small tetra­gonal crystals had formed. The anti­cipated thio­strepton crystal polymorph was in the space group *P*4_3_2_1_2 (PDB ID: 1e9w), with unit-cell constants *a* = 26.58, *b* = 26.58, *c* = 27.44 Å, α = 90, β = 90 and γ = 90° (Bond *et al.*, 2001 ▸).

### Instruments and data collection

Data were collected using a Talos F200C side-entry transmission electron microscope (Thermo Fisher Scientific) operating at 200 keV. The microscope optics were configured to deliver a low flux parallel beam on the sample and to collect selected area electron dif­frac­tion. Specifically, we used an extractor voltage of 4150 V, a gun lens of 4, spot sizes in the range 9–11, and a C2 aperture of 70 µm. For each configuration, a near-parallel beam was achieved by adjusting the C2 lens current to a value of ∼44.8% at spot size 11 and ∼45.8% at spot size 9, yielding a focused beam at the back focal plane of the objective lens; that plane was assumed to be coplanar with the objective aperture. These conditions yielded a beam approximately 3 µm in diameter, delivering an electron flux den­sity of ∼0.01 e^−^/Å^2^/s at spot size 11, 0.03 e^−^/Å^2^/s at spot size 10, and 0.045 e^−^/Å^2^/s at spot size 9. A 100 µm diameter selected area aperture was used to sample from a ∼1.2 µm radius circular area of the conjugate image plane. A virtual camera length setting of 420 mm yielded patterns that, as sampled by the Apollo detector, mapped a resolution of 0.8 Å at their edge. A virtual camera length setting of 670 mm yielded patterns captured by the Apollo detector with a resolution of 1.4 Å at their edge. Previous calibration of the TEM’s virtual camera length setting with a polycrystalline gold dif­frac­tion standard informed a correction of these virtual camera lengths to 540 and 860 mm, respectively, as the proper detector distance argument for subsequent crystallographic data processing. For com­parison, data was also acquired using a Ceta-D detector mounted in line with the Apollo, on the same Talos F200C microscope; dif­frac­tion was recorded under the same beam conditions as for the Apollo. Due to the different position of this detector relative to the specimen, a virtual camera length setting of 960 mm was necessary to capture patterns with a resolution of 0.8 Å at their edge. This was corrected in data processing to a 948 mm detector distance, as informed by the calibration detailed above. Diffraction was recorded as crystals were unidirectionally rotated at a fixed rate of speed, typically from 0.3 to 2° s^−1^. A standard tilt series spanned a 100° wedge of data, from +50 to −50°. All crystals were aligned to eucentricity such that they remained within the selected area aperture during the entire tilt range.

### Estimation of electron flux

Electron beam flux density estimates were measured for all selected area electron dif­frac­tion settings described in methods Section 2.2. Prior to any measurements, gain reference images were collected over vacuum with a flat field parallel beam in imaging mode encom­passing the entire detector and delivering approximately 25 e^−^/pix/s until a target of 4000 e^−^/pix was reached. Electron flux was estimated from counts in a gain-corrected image of the parallel beam acquired by the Apollo. The number of electrons measured per pixel was determined from raw counts using a conversion factor of 16 counts per electron, corresponding to the value assigned to each detected event in Apollo’s firmware. These values were used to determine the flux density as a function of spot size, corresponding to ∼0.01, 0.03, and 0.045 e^−^/Å^2^/s for spot sizes 11, 10, and 9, respectively. In a second estimate, we recorded the flu screen current readout obtained when exposed to the parallel beam at each setting. These current readings (in amperes) were divided by the charge of an electron (1.602 × 10^−19^ C) and the size of the illuminated area (in Å^2^) from each trial to achieve measures of flux density in e^−^/Å^2^/s. These were determined as 0.0252 e^−^/Å^2^/s for spot size 9 and 0.0147 e^−^/Å^2^/s for spot size 10. The screen current readout at spot size 11 was below the threshold of detection and read out as 0 nA. Given the notable disagreement between these two approximations, we deemed it likely that beam current readings by the fluorescent screen at low electron flux were underestimated, supported by the 0 nA reading at spot size 11, and so adopt flux density and fluence values as measured by the Apollo detector for this article.

### Calculated estimates of electron counts and coincidence loss in dif­frac­tion experiments

Throughout this article, units of e^−^/pix/s always refer to the detection (output) rate on the sensor, not the incidence (input) rate, since the detection rate may be lower than the incidence rate due to coincidence loss. To numerically simulate the number of incident electrons counted per pixel per second (e^−^/pix/s) during EBEC, we assumed that each electron event impinging on the detector had the potential to activate a cluster of adjacent pixels (see Fig. S1 in the supporting information). The Apollo detector applies this same logic during event detection, by considering blocks of up to 5 × 5 physical pixels during centroid-based electron event registration (Figs. S1–S3). When eight adjacent pixels, present side-by-side or diagonal, are simultaneously activated, a bounding box is defined with a maximum size of 5 × 5; the centroid of con­nected signal pixels within this box is assumed to represent a single incident primary electron. Each 5 × 5 block of physical pixels may successfully detect multiple incident primary electrons, provided that the activated pixels from each are not adjacent.

For each block of physical pixels on the Apollo sensor, the detection time inter­val is 418 µs. Therefore, the maximum counting rate of any pixel in isolation is 2392 e^−^/pix/s. However, because simultaneously activated adjacent pixels are assumed to represent the same incident primary electron, if one pixel is counting 2392 e^−^/pix/s, its eight adjacent pixels must necessarily be counting 0 e^−^/pix/s. Therefore, the maxi­mum average counting rate, averaged over a block of pixels is one-ninth the inter­nal counting rate, equating to ∼266 e^−^/pix/s. In practice, the fraction of incident electrons that activates more than one pixel will further reduce this maximum average counting rate. For example, a detection event consisting of two adjacent activated pixels has ten adjacent inactivated pixels. If all incident primary electrons always activated two adjacent pixels, then the maximum average counting rate would be 2392/(2 + 10) = ∼200 e^−^/pix/s. Of course, since the shape and size of detection events on the sensor span a range of possibilities, the maximum average counting rate will be a weighted average of all of these possibilities, with the result no more than 266 e^−^/pix/s.

**Figure 1:**
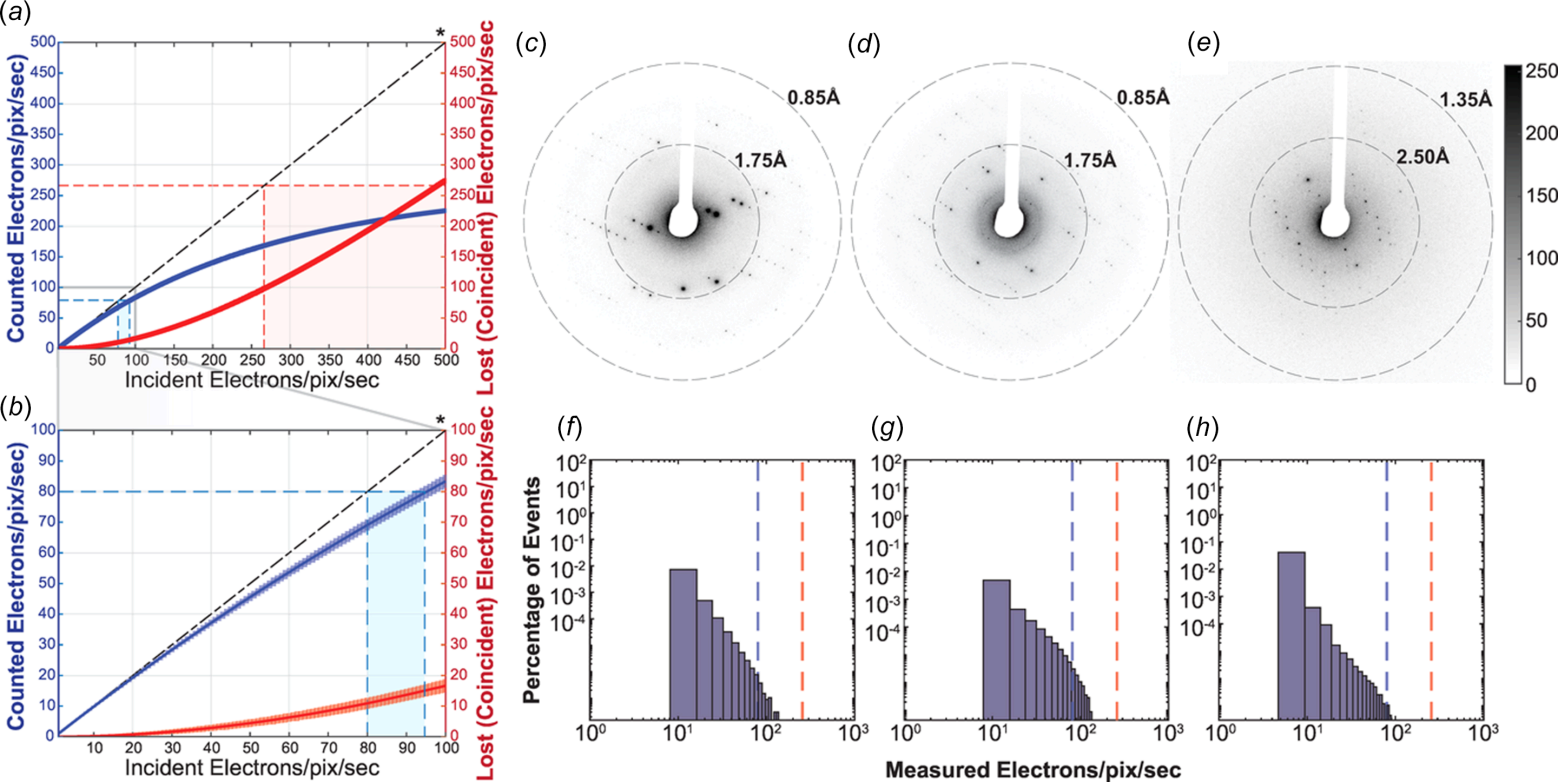
Analysis of EBEC dif­frac­tion data as a function of incident electron flux. (*a*) Simulated estimates of anti­cipated CL as a function of electron flux incident on a given detector pixel. Inset (*b*) magnifies the region from 0 to 100 e^−^/pix/s in part (*a*). An asterisk (*) denotes the hypothetical line corresponding to perfect counting. The maximum inter­nal count rate for the Apollo detector is denoted in bold: 2392 e^−^/pix/s. EBEC dif­frac­tion patterns from salen ligand crystals (*c*), biotin crystals (*d*), and thio­strepton crystals (*e*) were collected with an incident flux of 0.045 e^−^/Å^2^/s on each crystal; resolution rings are marked with dashed lines. Measured electron count distributions in EBEC MicroED data sets collected from crystals of the salen ligand (*f*), biotin (*g*), and thio­strepton (*h*) at the same incident flux are shown. Distributions tabulate all registered e^−^/pix/s greater than zero.

To evaluate the impact of coincidence loss due to overlapping detection events from multiple incident primary electrons, we performed numerical simulations in which nine independent arrays of virtual counts were generated sampling a random temporal distribution of electron arrival on the pixel within a one second inter­val, defined by an incident electron flux on a pixel. The nine independent arrays of incident electrons simulated a cluster of adjacent pixels. Each was sampled at the inter­nal count rate, and the coincidence of counts across all nine was assessed per second. Each coincident pair of events within a pixel or between pixels in a cluster contributed to the count of lost electrons. That process was sampled in 1000 trials, each with a random temporal distribution of electron counts per pixel (Script 1 in the supporting information). Averages and standard deviations were calculated and plotted for the measured e^−^/pix/s and the corresponding lost count of e^−^/pix/s (Fig. 1 ▸ and Figs. S1–S3).

While the simulated data is in agreement with anti­cipated parameters, it is important to note that experimental measures of coincidence loss are often greater than those simulated here. This is due to many factors, including the fact that non-uniform illumination can lead to local loss of electron counts due to hard limits on count rates during sensing. This is evidenced in dif­frac­tion data collected at increasing incident electron flux from dose-insensitive well-diffracting crystals of Co^II^*meso*-tetra­phenyl­porphyrin, which we used as a dif­frac­tion standard. In that case, an approximately fourfold increase in incident flux does not result in a fourfold increase in observed dif­frac­tion counts across all measured signal pixels (Fig. S4), and increasing flux density by a factor of 8.4, from ∼0.01 to ∼0.084 e^−^/Å^2^/s, showed a pronounced loss of counts at the brightest reflections. This was observed in scatterplots of counts at 0.01 e^−^/Å^2^/s *versus* higher incident flux values, and histograms of count ratios across those conditions [Figs. S4(*e*)–(*h*)]. Based on that experiment, we elected to limit our incident flux density for subsequent experiments to less than 0.05 e^−^/Å^2^/s.

### The Apollo detector and its use for MicroED EBEC data measurements

The Apollo detector houses a monolithic active pixel sensor performing correlated double sampling, on-chip thresholding, on-chip event detection, and event encoding. The sensor is com­posed of 4 × 2 rectangular sensor segments com­prising a contiguous 4096 × 4096 array of 8 µm pixels. The time resolution for event detection is 418 µs. Pixel readouts from the sensor are directly transferred to on-board field pro­gram­mable gate arrays (FPGA) that carry out super-resolution centroid mapping, yielding super-resolved dose-fractionated movie frames with 8192 × 8192 virtual pixels. In these images, each counted electron is converted to a signal value of 16 per pixel. Although the Apollo sensor is capable of a maximum average counting rate of ∼266 e^−^/pix/s, the bandwidth of the on-board memory in the Apollo camera limits the detection rate to a maximum of ∼126 million e^−^/sensor segment/s, which equates to a maximum average counting rate of ∼60 e^−^/pix/s or a total counting rate of >1 billion e^−^/s.

We note that these maximum counting rates are averaged over blocks of pixels and therefore are a straightforward limit during uniform illumination of the sensor. However, in dif­frac­tion, the illumination is highly non-uniform, with primary electrons concentrated in discrete reflections. In this case, the detected intensity of each reflection may be much higher than the counting rate limits discussed above, because pixels between reflections will have much lower detection rates. On average, the counting rate limits are still satisfied.

To record dif­frac­tion images from static crystals in electron counting mode, the Apollo detector was operated at an output dose-fractionated movie frame rate of 60 Hz, meaning that each output movie frame consists of the sum of all the events detected for ∼16.7 ms. A gain reference was performed by the protocol described in Section 2.3 first, and all subsequent dif­frac­tion images were gain-corrected and saved as full 8192 × 8192 frames. In this same mode, to facilitate electron counting for continuous rotation MicroED data collection, the detector was operated at a rate of 0.3–2 Hz, and stage rotation was configured such that each frame sampled one degree of data. The typical data set, under our fast data collection scheme, sampled 100° in a single 100 frame-movie saved in MRC format; this was converted to a series of individual SMV format frames for processing. Slow rotation (0.3°/s) data collection data sets sampled an equivalent wedge of reciprocal space, in an equivalent number of frames, but with a larger corresponding total fluence.

#### Using SerialEM for MicroED data collection

SerialEM was configured to record dif­frac­tion data on the Apollo detector using logic similar to that previously described by de la Cruz and co-workers (de la Cruz *et al.*, 2019 ▸). Briefly, three modes were configured to facilitate data collection using low-dose settings in SerialEM as follows: ‘View’ mode was used for generating montage overviews of the center working area of a grid. The typical montage was configured to sample an array of 7 × 7, 155× magnification images. These images were recorded with an exposure time of 0.25 s, 2× binned, yielding sufficient resolution to identify potential crystallites of inter­est (Fig. S5). ‘Record’ mode was used to corroborate the positions of crystals identified in the grid montage overview. To achieve this, it was configured to acquire real-space images at 2500× to 4300× magnification, with the C2 lens condensed to illuminate the same area as would be sampled by a parallel beam when sampling dif­frac­tion. The selected area aperture, not under the control of SerialEM, was inserted as needed to confirm each crystal remained eucentric within its bounds throughout the entirety of the tilt range used for data collection (±50°). ‘Search’ mode was configured to sample selected area dif­frac­tion as described in Section 2.2, with a virtual camera length of 420–670 mm. In this mode, the Apollo was set to record single 100-frame movies per data set. Target crystal locations were identified from the montage image, confirmed in ‘Record’ mode, and added to the navigator. A script (Script 2 in the supporting information) was then used to collect continuous rotation MicroED data from consecutive target locations, stored in the navigator.

### Analysis of reflection intensities in EBEC dif­frac­tion patterns

Diffraction patterns measured by the Apollo detector as 8192 × 8192 × 100 data stacks in MRC format were used to generate histograms of all counts, as well as counts associated with measured reflections. Reflections were detected by bandpass filtering individual frames in a dif­frac­tion image stack and selecting all pixels that were at least 2 or 3 standard deviations above the measured background value. That threshold depended primarily on the level of background signal produced primarily by inelastic and incoherent scattering produced by the sample and substrate. These pixels were considered in the set of all associated with reflections for subsequent counting analyses and were also used to count individual reflections *via* an image segmentation routine that partitioned connected sets of pixels into individual clusters; each cluster was assumed to be a single reflection. All statistics quoted for reflections in a given data set were determined based on these subsets, and all figures displaying maximum or summed intensity dif­frac­tion patterns show this subset of selected pixels.

### Reduction and processing of MicroED Data

MicroED movies collected on the Ceta-D camera were binned into frames of size 2048 × 2048 pixels, saved in SER file format, and then converted to SMV image stacks using the script ser2smv (Hattne *et al.*, 2015 ▸). Frames in these stacks were reduced in *XDS* (Sheldrick, 2008 ▸), enforcing a corrected virtual camera length of 948 mm. Data were indexed first without enforcement of unit-cell constants or space-group symmetry to validate they were of sufficient quality for analysis, then reprocessed while enforcing the expected unit cell and space-group symmetry for each com­pound. Reflections were integrated to 0.8 Å resolution, to match the resolution considered for reflections measured from the Apollo camera, then scaled in *XSCALE*.

EBEC MicroED movies collected on the Apollo DED were converted from MRC file format to SMV image stacks with custom scripts run on MATLAB Version 2023b. During file conversion, frame sizes were reduced to 2048 × 2048 pixels, and a value of 1 was added uniformly to every pixel in each frame to avoid pixel values of 0 in the SMV images. Data collected using a virtual camera length of 420 mm were processed in *XDS* enforcing a corrected detector distance of 540 mm. Thio­strepton data collected using a virtual camera length of 670 mm were processed using a corrected detector distance of 860 mm. Data were again indexed first without enforcement of unit-cell constants or space-group symmetry to validate that they were of sufficient quality for analysis, then reprocessed while enforcing the expected unit cell and space-group symmetry for each com­pound. For the salen ligand and biotin crystals, reflections were integrated to a resolution of 0.8 Å, then scaled in *XSCALE.* For thio­strepton crystals, reflections were integrated to a resolution of 1.5–2.0 Å, then scaled as described previously. All data reduction statistics are reported as calculated by *XSCALE*.

### Coincidence loss intensity adjustment and processing of EBEC MicroED data

We sought to determine whether a CL adjustment would improve EBEC data. We calculated the mean loss of electrons for a given rate of measured counts in e^−^/pix/s (Fig. 1 ▸) and systematically added these counts to every pixel that measured between 10 and 260 e^−^/s in dif­frac­tion frames. Electron counts were deduced from raw gain-corrected intensity values as described in Section 2.6, assuming a conversion factor of 16 counts per electron, and normalizing for the integration time per frame; where frames were typically recorded at a rate of 2 or 0.3 Hz. In this scheme, a pixel with a measured count of 10 e^−^/s received < 0.2 additional e^−^/s, while ∼25 e^−^/s were added to pixels with a measured count of 100 e^−^/s (Fig. S3). These values are conservative, given that real CL percentages are likely higher in experimental data, as indicated by Nakane *et al.* (2020 ▸). This addition of electron counts was systematically performed for each of the 8192 × 8192 pixels in all 100 raw super-resolution dif­frac­tion images of an EBEC MicroED data set. The counts-adjusted images were once again saved in MRC format for subsequent processing (Script 3 in the supporting information). CL-adjusted dif­frac­tion movies were converted into individual SMV frames as described previously in Section 2.7, and used for subsequent data reduction in *XDS*.

### Structure determination and refinement

**Figure 2:**
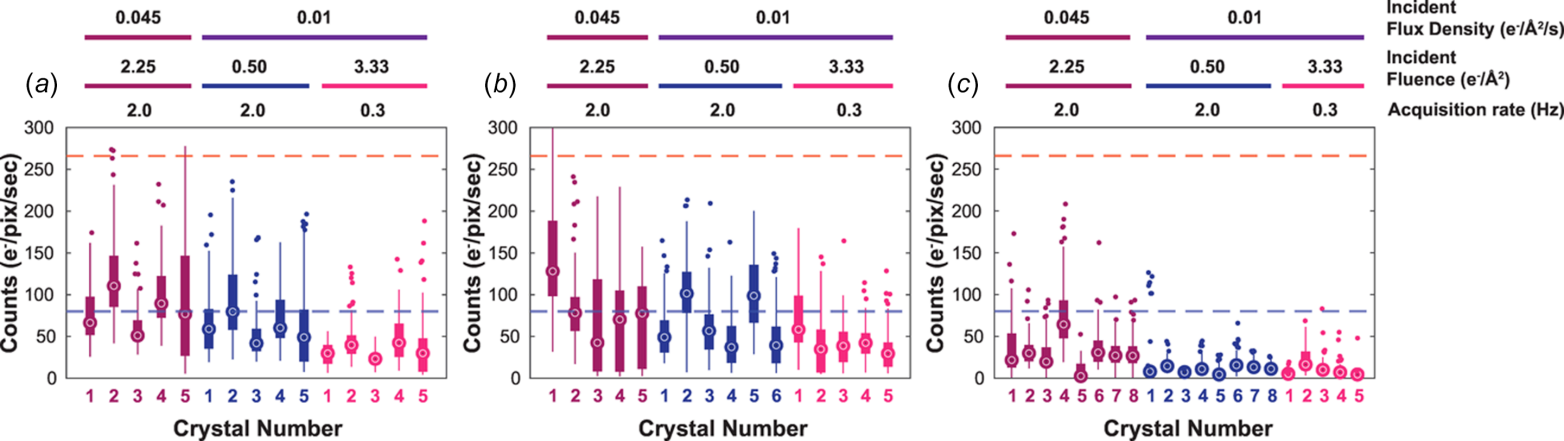
Analysis of measured counts within Bragg reflections in EBEC MicroED data sets with different incident beam fluence. Crystals were exposed to three different levels of total incident beam fluence: 0.5 (blue), 2.25 (magenta), and 3.33 e^−^/Å^2^/s (pink). Box plots show the distribution of counts in measured reflections for each crystal of salen ligand (*a*), biotin (*b*), and thio­strepton (*c*). For each crystal, an open circle shows the median, boxes show the bounds of the upper and lower quartiles, and dots mark outliers. A dashed blue line marks 80 e^−^/pix/s, while a red dashed line marks 266 e^−^/pix/s.

*Ab initio* phasing of the salen ligand and biotin structures was performed using *SHELXD* or *SHELXT* from HKL files reduced by *XDS* (Sheldrick, 2008 ▸). The resulting atomic co­ordinates were then further refined in *SHELXL* (Hübschle *et al.*, 2011 ▸; Sheldrick, 2015*b* ▸) as follows: structures obtained from *SHELXD*/*SHELXT* were refined with a WGHT parameter of 0.2 over batches of 1000 cycles of least-squares refinement until the *R* factors converged, using electron scattering factors parameterized as four Gaussians for each element (Saha *et al.*, 2022 ▸). Missing non-H atoms were assigned guided by evident Q-peaks from the ‘find Q-peaks’ module in *SHELXL*. H atoms were placed when evident in the *F*_o_–*F*_c_ map and Q-peaks, and when appropriate based on likely mol­ecular geometry, but were omitted during refinement for the purposes of com­parison between data sets. All refinements were performed treating *B* factors as isotropic. For each sample, representative structures were determined with riding H atoms and anisotropic *B*-factor refinement whenever doing so did not result in non-positive definite (NPD) *B* factors on any atoms (Fig. 2 ▸). Structures of thio­strepton were determined by mol­ecular replacement using *MOLREP* with PDB entry 1e9w as a search model and refined in *PHENIX*. Bond-length restraints of 1.7 ± 0.02 Å were implemented for all five thia­zole S atoms and their neighboring backbone C atoms to keep the thia­zole rings intact during cycles of refinement. Planarity restraints, with a standard deviation of 0.005 Å, were also applied over each atom in an *sp*^2^-hybridization environment on the thia­zole rings of the thio­strepton mol­ecule. For each thio­strepton data set used for structure determination, mol­ecular replacement in *MOLREP* was also performed using the model from PDB entry 1e9w with all residues mutated to alanine (Vagin & Teplyakov, 1997 ▸), followed by a cycle of rigid-body refinement in *PHENIX* (Adams *et al.*, 2010 ▸), to visualize Fourier difference maps to reveal density for atoms not supplied in the search model. Isotropic *B* factors were refined over residues. Structures were visualized in *Coot* and rendered in *PyMol*. Mol­ecular structure diagrams were generated in *ORTEP-3* (Farrugia, 2012 ▸) using CIF files generated from *SHELX* refinement.

## Results

### Coincidence loss estimates when applying fast EBEC to electron dif­frac­tion

Coincidence loss presents a major challenge for electron counting since it reduces the linearity of the sensor response and decreases the effective dynamic range achievable by that sensor. In dif­frac­tion measurements, the undercounting of coincident electrons decreases signal at bright reflections, leading to inaccurate integration of intensities. While single-particle imaging experiments can rely on a relatively constant illumination profile on the detector for estimates of coincidence, one cannot readily determine the anti­cipated degree of non-zero coincidence at a given Bragg reflection by simply knowing the incident flux on the crystal being diffracted. This makes it imperative to anti­cipate the degree of CL expected at any given pixel under any possible electron flux at that pixel.

The likelihood of zero coincidence can be estimated for a single pixel in a counting detector. For a given count rate *M* and flux *N*, this can be determined by combinatorics as *P*(*M*,*N*) = [*M*!/(*M* – *N*)!](1/*M^N^*), for *M* > *N* (Hattne *et al.*, 2023 ▸). Similar models have been applied to describe the impact of CL on detector quantum efficiency in both electron imaging and spectroscopy experiments in the context of a variety of direct detectors (Li *et al.*, 2013 ▸; McMullan *et al.*, 2014 ▸; Hart *et al.*, 2017 ▸). The probability indicates that deviations from zero coincidence would be expected despite high count rates and low flux values, requiring careful consideration of the chosen flux for an experiment. This is true if each detector pixel is considered to count with full independence of all others, and more so if events across neighboring pixels are considered correlated during counting (Fig. S1). More specifically, given that each pixel on the Apollo sensor counts at a rate of 2392 e^−^/pix/s, we can first consider the condition where electrons arrive only at a single pixel and it counts them independently of all other pixels on the sensor. Under these conditions, a 3% CL is expected when detecting a true incident flux of 100 e^−^/pix/s; a 10% CL is expected for a true incident flux of 500 e^−^/pix/s (Fig. S2). However, in a more realistic scenario, each electron strikes a cluster of pixels on the sensor, and counts are assessed from 3 × 3 patches of pixels on the sensor. Simulating that indicates a lower bound of ∼18% CL is anti­cipated for an incident flux of 100 e^−^/pix/s, and >50% CL is expected for an incident flux of 500 e^−^/pix/s (Fig. S3).

Correlation between adjacent pixels during counting means the Apollo, with an inter­nal count rate of 2392 e^−^/pix/s, has an effective counting rate of ∼266 e^−^/pix/s. Given that effective count rate, a true incident flux of 96 e^−^/s on a pixel would result in only ∼80 e^−^/s being counted [Figs. 1(*a*)–(*b*) ▸]. Con­versely, if less than 80 e^−^/pix/s are detected, CL is expected to be lower than 20%. For example, if 10 e^−^/pix/s are detected, an average loss of less than 1 e^−^/pix/s is expected [Figs. 1(*a*) ▸–(*b*) ▸]. These calculations set important bounds for signal counts at reflections and, ultimately, for incident beam flux on a crystal. When diffracting from small-mol­ecule crystals, we therefore targeted a maximum measured count rate of 80 e^−^/pix/s and set out to define experimental conditions yielding reflection counts that obeyed this limit when diffracting from the salen ligand, biotin, and thio­strepton crystals [Figs. 1(*c*)–(*h*) ▸].

### The impact of electron beam flux on fast EBEC for small-mol­ecule electron dif­frac­tion

To assess the degree of CL observed in EBEC MicroED patterns, we initially recorded dif­frac­tion from six salen ligand crystals at increasing values of incident electron beam flux density on the crystal: ∼0.01, 0.03, and 0.045 e^−^/Å^2^/s (Fig. S6). We chose salen ligand crystals since their unit cell, mor­phology, and degree of order were characteristic of the type of organic small-mol­ecule microcrystals that might yield *ab initio* structures by MicroED. We recorded 1 s-long 60-frame movies under these conditions, noting that the illuminated crystals were stable and did not suffer any radiation-induced decay in dif­frac­tion signal during that exposure. Electron count distributions from all measured reflections detected in second-long movies from various crystals showed maximum counts ranging from 71 to 206 e^−^/pix/s for an incident flux density of 0.01 e^−^/Å^2^/s, and maximum counts ranging from 8 to 246 e^−^/pix/s for the highest incident flux density of 0.045 e^−^/Å^2^/s (Fig. S6 and Table S1). These measurements underscored the uncertainty of maximal electron counts at reflections as a function of incident beam flux on a crystal, which is affected by shot noise. However, the fraction of pixels in measured reflections that exceeded 80 e^−^/pix/s more closely mirrored the changes in incident beam flux (Table S1). This inspired a further analysis of CL and its impact on MicroED data quality.

We assessed the impact of EBEC data collection on the overall quality of MicroED data collected with settings typically used for small-mol­ecule *ab initio* structure determination (Figs. S7–S9). To assess the quality of EBEC data with respect to conventional data sets, we directly com­pared dif­frac­tion from the salen ligand crystals, measured using either the scintillator-based CMOS-based camera (Ceta-D) or the Apollo detector. Diffraction movies were recorded with both the Ceta-D and the Apollo from individual salen ligand crystals with incident electron beam flux densities of 0.01 and 0.045 e^−^/Å^2^/s. EBEC data showed improved contrast and signal-to-noise (Figs. S7–S9), but, under these conditions, a large fraction of measured pixels in EBEC patterns had counts in the range 10–80 e^−^/pix/s, where some CL might be expected (Fig. S9).

### The impact of fast EBEC on the quality of small-mol­ecule MicroED data sets

To determine whether the CL observed during fast EBEC data collection would impact structure determination, we analyzed data under varying illuminating beam fluence from crystals of our three candidate mol­ecules: the salen ligand, biotin, and thio­strepton. For all three samples, we cataloged the counts of e^−^/s for every pixel in each of the 100 frames of a measured data set (Fig. 2 ▸ and Figs. S10–S12). Distributions of electron counts showed that the majority of pixels received only a few e^−^/s, including at our highest chosen illuminating flux density of 0.045 e^−^/Å^2^/s [Figs. 1(*f*)–(*h*) ▸ and 2 ▸]. Low counts were generally observed, even from the salen ligand crystals, which were the strongest diffracting and most robust of our chosen samples [Figs. 1(*f*) ▸ and 2(*a*) ▸, and Fig. S10]. However, in some high flux (0.045 e^−^/Å^2^/s) salen ligand data sets, as many as 0.03% of pixels in a given dif­frac­tion frame had counts above 80 e^−^/pix/s [Figs. S10(*c*)–(*d*),(*g*)–(*h*),(*k*)–(*l*)]. By com­parison, salen ligand dif­frac­tion collected at our lowest chosen flux of 0.01 e^−^/Å^2^/s had fewer pixels with signal above this threshold [Figs. S10(*c*)–(*d*),(*g*)–(*h*),(*k*)–(*l*)].

Equivalent count distributions were observed for biotin crystal reflections [Figs. 1(*g*) ▸ and 2(*b*) ▸, and Fig. S11], although some of those reflections still registered electron counts above the 80 e^−^/pix/s threshold [Figs. S11(*c*)–(*d*),(*g*)–(*h*),(*k*)–(*l*)]. In contrast, under equivalent conditions, thio­strepton crystals did not diffract as brightly or to atomic resolution; they consistently yielded ∼1.5–2 Å data­sets even at the highest incident beam flux density. Counts in the thio­strepton crystal data were also on average 2–5 times lower than those from the biotin or salen ligand crystals and rarely exceeded 80 e^−^/pix/s [Figs. 1(*h*) ▸ and 2(*c*) ▸, and Fig. S12]. Those counts are consistent with the com­paratively lower total number of illuminated unit cells and overall lower dif­frac­tion quality of the thio­strepton crystals.

Reasoning that a CL-induced reduction of the dynamic range of measured intensities might be detected as pseudo-twinning, we charted data reduction statistics and, in particular, the twinning-indicator L-test result for EBEC MicroED data (Figs. S13 and S14). All salen ligand and biotin crystals rotated at 0.3°/s yielded data for which the L-test determined a twin fraction of 0. In contrast, more quickly rotating salen ligand and biotin crystals had L-statistic-derived estimated twin fractions that, while low, were greater than zero on average (Table S7). This indicated that fast EBEC sampled from strongly diffracting crystals might suffer a mild degree of CL that can be registered by twin tests (Fig. S14). This trend in degree of pseudo-twinning as indicated by the L-test was mirrored in correlations between observed structure factors with a reference set of calculated structure factors for salen ligand data sets acquired under each fluence condition, where weaker correlations were measured under conditions that gave greater estimated twin fractions (Fig. S19).

### Leveraging fast EBEC for low-dose MicroED structures of beam-sensitive organic small mol­ecules

**Figure 3:**
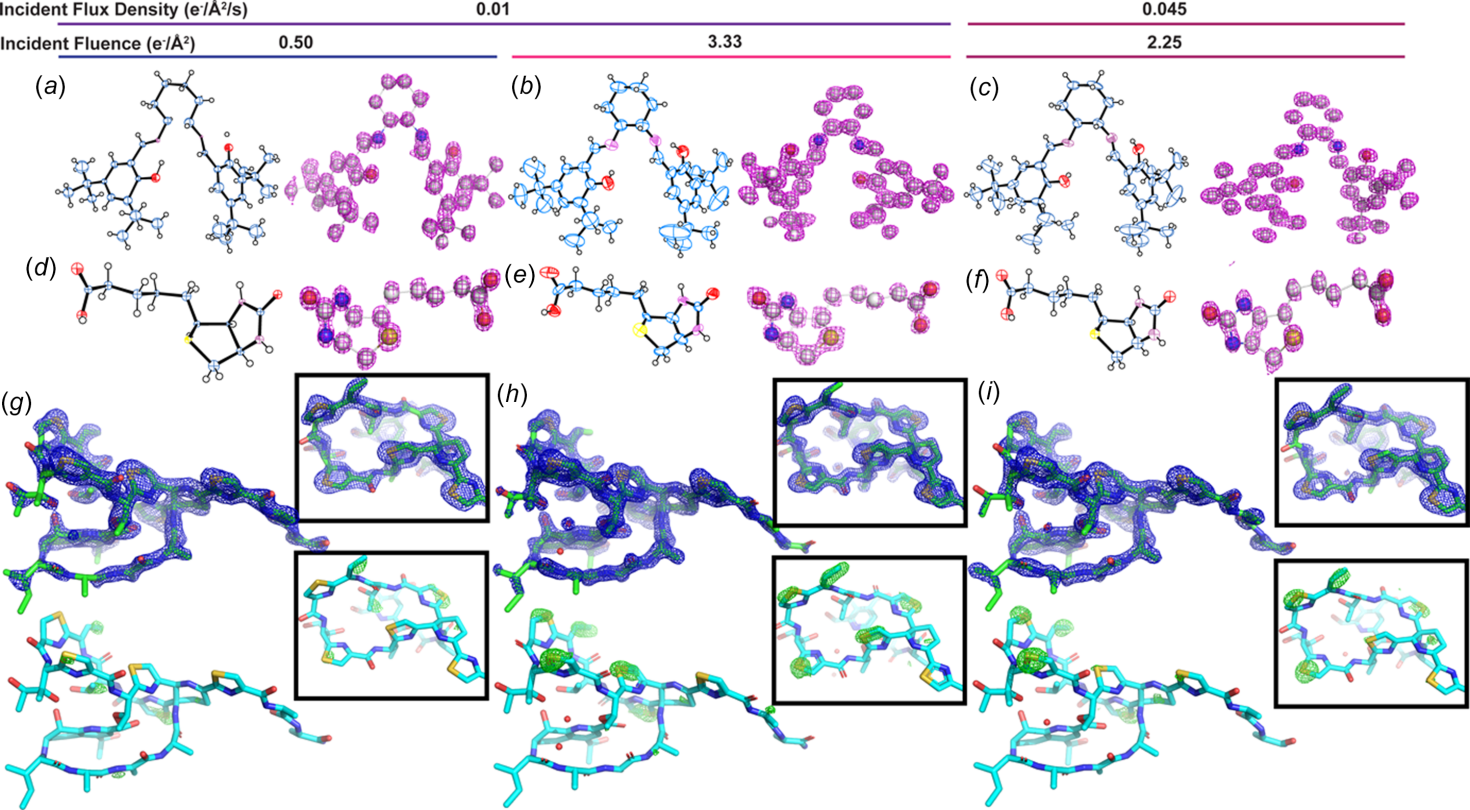
Structures of salen ligand (*a*)–(*c*), biotin (*d*)–(*f*), and thio­strepton (*g*)–(*i*) determined from EBEC MicroED data collected with different incident electron beam fluences. For both salen ligand and biotin mol­ecules, shown are *ORTEP* diagrams and 3D models with the *F*_o_ density map contoured at 2σ. Single crystals were exposed to a total fluence of 0.5 [2°/s rotation, 2 integrated frames/s, 0.01 e^−^/Å^2^/s flux density, parts (*a*), (*d*), and (*g*)], 3.33 [0.3°/s rotation, 0.3 integrated frames/s, 0.01 e^−^/Å^2^/s flux density, parts (*b*), (*e*), and (*h*)], and 2.25 e^−^/Å^2^ [2°/s rotation, 2 integrated frames/s, 0.045 e^−^/Å^2^/s flux density, parts (*c*), (*f*), and (*i*)]. H atoms were included in the refinement and are displayed in the *ORTEP* diagrams, but were excluded from ball-and-stick models for clarity. Atomic *B* factors were refined anisotropically when possible, as was permitted for the 3.33 e^−^/Å^2^ fluence trials on the salen ligand and biotin, and the 2.25 e^−^/Å^2^ fluence trial on the salen ligand. Likewise, higher fluence generally allowed for greater accuracy in bond lengths for these com­pounds. Structures of thio­strepton are drawn as green models with superimposed blue 2*F*_o_–*F*_c_ maps contoured at 1.8σ. Each is determined from a single crystal to 2.0 Å [part (*g*)], 1.5 Å [part (*h*)], and 1.8 Å resolution [part (*i*)]. Beneath each is the same model (cyan) superimposed with a green *F*_o_–*F*_c_ map at 3σ, calculated from rigid-body refinement of the measured data against a poly-alanine model of thio­strepton. The clearest definition for side chains in the electrostatic potential map, and likewise the most prominent *F*_o_–*F*_c_ density in the poly-alanine trial, was achieved with the highest fluence, the 3.33 e^−^/Å^2^ condition.

**Figure 4:**
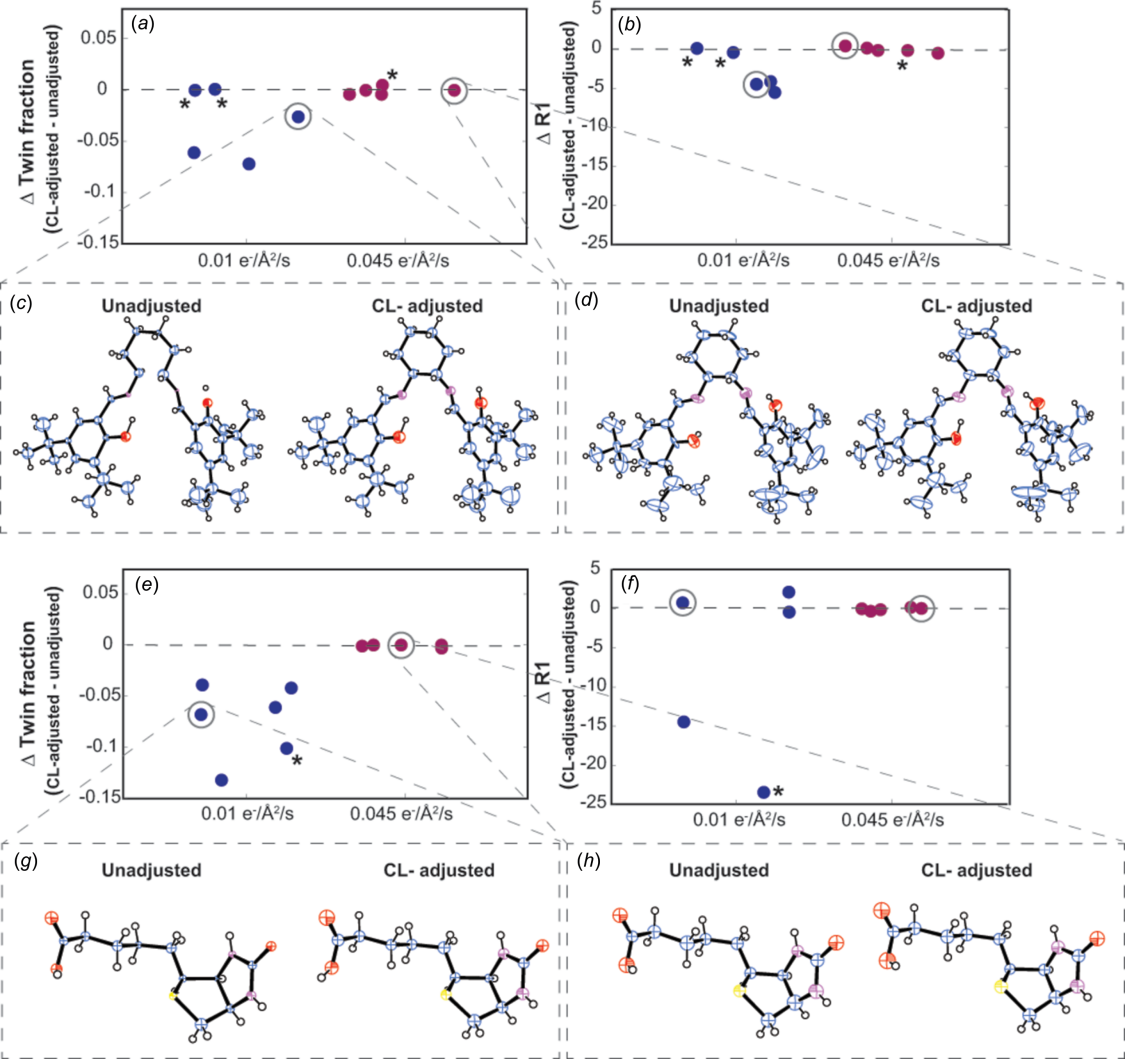
Analysis of CL-adjusted EBEC data collected at 2° s^−1^ rotation and 2 frames per second integration. Change in estimated twin fraction (*a*)/(*e*) and achievable *R*1 (*b*)/(*f*) from the refinement for fast EBEC data sets as a result of CL adjustment, from crystals of the salen ligand (*a*)–(*d*) and biotin (*e*)–(*h*). Points denote data acquired with an incident flux density of 0.01 e^−^/Å^2^/s (blue) and data sets acquired with an incident flux density of 0.045 e^−^/Å^2^/s (magenta). Small mol­ecule structures determined from data with and without CL-adjustment are shown for representative points, outlined in gray for salen ligand (*c*)/(*d*) and biotin (*g*)/(*h*). Anisotropic refinement of ADPs was performed where it did not result in ADP refinement to non-positive-definite (NPD) values. Asterisks [parts (*a*), (*b*), (*e*) and (*f*)] denote data sets for which refinements were unstable and did not yield a suitable refined structure.

We evaluated the ability to determine accurate structures of small mol­ecules from EBEC MicroED data collected using an incident electron beam flux density of only 0.01 e^−^/Å^2^/s. This flux density yielded high-quality data sets from all sampled crystals; data were sufficiently accurate and com­plete to yield *ab initio* structures from salen ligand and biotin crystals (Fig. 3 ▸ and Tables 1 ▸ and 2 ▸). Preliminary solutions from salen ligand data­sets recorded under these conditions contained 40 accurately placed atoms that could be further refined to structures with an average *R*_1_/*wR*_all_ of 0.2689/0.2928 and an average GooF of 1.989 and showed clear density for two H atoms [Figs. 3(*a*)–(*c*) ▸]. Similarly, preliminary solutions obtained from biotin crystals showed 16 atoms and could be further refined to structures with an average *R*_1_/*wR*_all_ of 0.2873/0.3244 and an average GooF of 1.590; these also showed clear density for two H atoms [Figs. 3(*d*)–(*f*) ▸]. Data sets that could not be stably refined using *SHELXL* had *R*_1_ statistics well exceeding 50% and were not included in subsequent analyses (Fig. 4 ▸). The representative data sets used for structure determination of the salen ligand and biotin under each condition were further com­pared by rescaling only the reflections commonly observed across all three trials and cataloguing the resulting statistics (Tables S3 and S4).

Data from the thio­strepton crystals failed to reach atomic resolution but were sufficient for mol­ecular replacement [Figs. 3(*g*)–(*i*) ▸ and Table 3 ▸]. Solutions could be achieved using *MOLREP* for data sets acquired from thio­strepton under all three fluence conditions, but subsequent refinement was most successful for crystals exposed to the highest fluence of 3.33 e^−^/Å^2^. A representative structure determined under these conditions could be refined to *R*_work_/*R*_free_ of 0.2026/0.2167, had an overall *B* factor of 13.09 Å^2^, and showed fully intact side-chain density for six residues, including all five thiazole rings on the molecule (see Table S6). Data collected at the lowest fluence (0.5 e^−^/Å^2^) were not generally suitable for high-quality refinement but were still sufficient to visualize the most ordered core of the mol­ecule. In these refinements, side-chain density was visible in Fourier difference maps resulting from the refinement of the data against poly-alanine models of thio­strepton, most prominently from data collected at the highest fluence [Figs. 3(*g*)–(*i*) ▸ and Table S2]. These ob­servations held when refining only the commonly observed reflections over all three trials, with the same set of *R*_free_ flags, against the same rigid-body models of thio­strepton and polyA-thio­strepton (Tables S5 and S6, and Fig. S13).

### A coincidence loss adjustment for EBEC-mediated MicroED and its impact on the accuracy of small-mol­ecule structures

To determine the potential benefit to be gained from reduced CL in fast EBEC MicroED data, we used the known counting rate of the Apollo detector and the measured electron counts per second for any given pixel to implement a simple CL adjustment. While limited, we hoped the adjustment might approximate closer to true counts from measured values and indicate whether more refined adjustments would be beneficial. The adjustment is determined from estimates of the number of undercounted electrons from numerical simulations [Fig. 1 ▸(*a*) and Figs. S1–S3]. As the rate of incoming electrons increases, the number of counts detected per unit time asymptotically approaches the effective maximum count rate per pixel. We estimate that, for the Apollo, this value should be ∼266 e^−^/pix/s. Based on this rate, numerical calculations would suggest that 80 counted e^−^/pix/s should be adjusted to ∼96 e^−^/pix/s, to account for CL. Although an ∼2 e^−^/pix/s standard deviation is associated with this correction, that degree of uncertainty is lower than the magnitude of the error due to potentially lost counts [Fig. 1 ▸(*a*) and Fig. S3]. These calculated adjustments were applied to raw measured pixel counts, creating CL-adjusted data sets with increased electron counts.

We applied the CL adjustment to all our EBEC MicroED data sets. We found that in fast EBEC dif­frac­tion patterns measured from salen ligand crystals illuminated with a flux density of 0.045 e^−^/Å^2^/s, approximately 0.03% of all pixels had counts greater than 80 e^−^/pix/s (Fig. S10). Despite the low number of pixels affected, those pixels were distributed across a wide number of frames and reflections. Some affected pixels had counts that approached the effective counting limit of the sensor (Fig. S10); these were principally observed in high-incident beam flux data sets. In contrast, data from crystals illuminated with the lowest beam flux had few or no pixels above this threshold. These trends were also displayed by data collected from biotin crystals (Fig. S11) and, to a far lesser degree, thio­strepton crystals, which diffracted weakly regardless of incident beam flux (Fig. S12).

To determine the potential impact of these CL-affected pixels on data reduction and structure determination, we com­pared raw unmodified dif­frac­tion intensities to their CL-adjusted counterparts. Data reduction parameters optimized for raw un-adjusted dif­frac­tion frames were used unchanged for processing of their CL-adjusted counterparts. CL adjustment of dif­frac­tion frames improved their dynamic range, particularly for low-resolution reflections (Figs. S16–S18). The consistency and accuracy of adjusted reflections were judged by data reduction statistics (Fig. 4 ▸, and Figs. S14 and S15). Analysis of twin law tests for unmodified and CL-adjusted EBEC MicroED data from salen ligand and biotin crystals illuminated with low fluence showed uniformly reduced evidence of pseudo-twinning after CL adjustment, consistent with its anti­cipated improvement of dynamic range (Fig. 4 ▸ and Table S3). However, applying the CL adjustment measurements at higher flux density (0.045 e^−^/Å^2^/s), the salen ligand and biotin crystal data sets generally yielded negligible changes to the estimated twin fraction. In most of these cases, CL adjustment decreased the estimated twin fraction, albeit slightly, though in the cases of three salen ligand crystals illuminated at 0.045 e^−^/Å^2^/s for 2.25 e^−^/Å^2^ total fluence we did observe negligible increases, in the range 0–1.1%, in the estimated twin fraction upon applying the CL adjustment. For salen ligand crystals illuminated at 0.01 e^−^/Å^2^/s for 0.5 e^−^/Å^2^ total fluence, we only observed one crystal that showed any increase, in this case of 0.01%, in its estimated twin fraction upon applying the adjustment (Table S7). For representative salen ligand data sets acquired at 2 Hz at each incident flux density condition, correlations between the observed structure factors and a reference set of calculated structure factors were likewise slightly improved following CL adjustment [Figs. S19(*a*) and S19(*c*)].

Using the L-test as a diagnostic of the degree of CL in MicroED data, we concluded that EBEC data collected quickly are generally improved by CL adjustment, but little improvement is seen for data acquired more slowly with higher total fluence. Notably, while CL adjustment did not meaningfully change *R*_merge_ and *I*/σ, structures from CL-adjusted data generally refined to lower *R*_1_/*wR*_all_ than their unadjusted counterparts (Fig. 4 ▸). Overall, these metrics indicate that more robust CL adjustments might further enhance the effective dynamic range of low-flux fast EBEC data and improve the quality of MicroED structures. These might, for instance, take into account the number of electrons counted at neighboring pixels when determining the degree of loss likely to have occurred at a given pixel position over a set time inter­val, rather than considering the expected loss to vary only as a function of incident flux at a single pixel. Ultimately, a more robust and thorough CL model would be needed for universal CL adjustments.

## Discussion and conclusions

<!?tlsb=-0.02pt>Electron counting is meant to allow a less obstructed view of signal close to the noise floor. However, despite its successful application to imaging in cryoEM, electron counting has been less widely adopted for MicroED. This is due to a variety of reasons, including the greater cost of counting detectors and fears that they might be damaged by the high electron flux at strong reflections during MicroED collection. It is also likely due to the risk of CL limiting the dynamic range of the data and limiting the feasibility and accuracy of structure solution. Nonetheless, a handful of structures of peptide and protein crystals have been determined by MicroED from data collected on DEDs (Gallagher-Jones *et al.*, 2020 ▸; Hattne *et al.*, 2019 ▸, 2023 ▸) in experiments engineered to reduce the likelihood of CL. However, those efforts are not expected to readily translate to accurate small-mol­ecule MicroED data collection.

We set out to (i) assess the feasibility of electron counting applied to small-mol­ecule MicroED data collection, (ii) determine the potential benefit of fast EBEC for the detection of accurate diffracted intensities from small-mol­ecule crystals by MicroED, and (iii) evaluate the impact of CL and a CL adjustment on small-mol­ecule MicroED. Compared to the Ceta-D, the detector of record in a large fraction of the deposited MicroED structures in the PDB, EBEC data collection facilitated more rapid data acquisition, thereby yielding high-com­pleteness single-crystal dif­frac­tion data sets that were less impacted by beam-induced radiolytic damage. Attempts to acquire data as rapidly using the Ceta-D, matching the stage rotation speed (2°/s) and frame rate (2 frames/s) used in the fast EBEC experiments, yielded data with inferior reduction statistics. In particular, for data collected on the salen ligand with an incident beam flux density of 0.01 e^−^/Å/s and fast rotation of 2°/s, overall *R*_merge_ statistics from these Ceta-D data sets were more than double what was achieved with fast EBEC (Table S4). For this com­parison, overall *I*/σ statistics were twice better using fast EBEC. That discrepancy was less dramatic when the same com­parison was made with a higher incident beam flux of 0.045 e^−^/Å/s (Table S5). Nonetheless, data reduction statistics from fast EBEC data were preferable in all cases.

High-quality fast EBEC data showed some degree of CL, but were sufficient for accurate *ab initio* structure determination by MicroED. Not surprisingly, the greatest dynamic range was observed under the highest beam fluence, granted by extended data collection times. Most importantly, however, accurate structures could be determined from fast rotation data with higher beam flux, where the measured electron counts were overall higher. Ultimately, optimizing the quality of dif­frac­tion movies involved balancing incident flux and speed of data collection to yield the greatest dynamic range in accurately measured intensities. Although considerable CL is expected for the pixels with measured counts approaching the effective count rate of the Apollo (∼266 e^−^/pix/s), we also note that this assumed count rate depends on the uniform propensity for electrons to impact pixels on a sensor. A higher count rate could be possible if that likelihood were skewed. This would be the case, for example, where a train of electrons only impinges on a single pixel and never its neighbors, a condition that may be present at Bragg reflections.

As the strong inter­action between the electron beam and microcrystals promises that dynamical and inelastic scattering will impact any measured MicroED data, it should also be noted that these effects remain a variable in optimizing EBEC data collection for small mol­ecules. Inelastic scattering distributes electrons that may have otherwise contributed to Bragg reflections randomly, such that they may arrive anywhere on the detector, with greater probability at low scattering angle, and therefore reduces risk of CL. Dynamical scattering redistributes electrons across reflections bound by the Bragg condition, which also decreases the effective dynamic range present in the measured reflections. If significant dynamical dif­frac­tion impacts the data, the likelihood of undercounting electrons at strong reflections due to CL is reduced; however, dynamical scattering would be expected to produce a similar effect to CL on intensity statistics diagnostics such as the L-test, and might be another reason pseudo-twinning is detected in a given data set. As we rarely see distinct evidence of strong dynamical scattering in MicroED tilt series on mol­ecular crystals containing primarily light atoms, such as violations of systematic absences or strong violations of Friedel’s Law, we assume that any pseudo-twinning detected by the L-test in this article is due to CL, but this possible ambiguity should be noted.

Dynamic range limitations might be circumvented by acquiring longer or multiple exposures, increasing the chances of incident electrons falling within the dynamic range of the detector, but such strategies likely increase the dose on target crystals. Alternatively, numerical estimates of CL can allow for adjustments that com­pensate for lost electrons to further enhance the dynamic range of measured dif­frac­tion patterns *ex post facto*. We found such an adjustment to slightly improve MicroED data collected from salen ligand and biotin crystals. Given the characteristics of these crystals, improved CL adjustments could generally improve EBEC MicroED data collected from small-mol­ecule crystals. DEDs could also fully overcome electron-counting limitations by operating at substanti­ally faster readout rates. For example, the readout of pixels in a detector operating at many kilohertz, as is achieved by the 4D Camera, would dramatically reduce CL, but would also produce large volumes of information to be handled *ex post facto* (Ercius *et al.*, 2023 ▸). Collectively, our experiments indicate that current fast EBEC approaches are sufficient for the determination of accurate *ab initio* structures by MicroED, with future speed improvements and CL adjustments continuing to reduce CL and improve data quality.

## Concluding remarks

With the growing inter­est in applying DEDs to dif­frac­tion measurements, the use of fast EBEC strategies offers advantages for MicroED data collection. We find that these tools enable the determination of accurate atomic structures of organic small mol­ecules with electron fluences as low as 0.5 e^−^/Å^2^. Although CL is expected in EBEC data, the count rates observed under low flux conditions suggest only a small fraction of diffracted intensities suffer significant losses. Further, a CL adjustment to measured electron counts can take into account anti­cipated losses of electron counts and enhance dynamic range. Importantly, fast EBEC expedites the determination of accurate structures from beam-sensitive biomolecules such as biotin, without imposing added labor or time to data collection. This is further facilitated by the com­patibility of new DEDs, such as the Apollo, with semi-automated data collection tools, such as SerialEM. Finally, by reducing the need for sampling and combining data from large numbers of crystals, fast EBEC further expands access to structures from rare beam-sensitive crystals, polymorphs, or trace impurities.

## Supplementary Material

PDB reference: 9cq0

Crystal structure: contains datablock(s) a, a_1, global. DOI: 10.1107/S2053229624012300/yp3239sup1.cif

Scripts, tables and figures. DOI: 10.1107/S2053229624012300/yp3239sup2.pdf

CCDC references: 2370186, 2370185

## Figures and Tables

**Table 1:** Structures from salen ligand single crystals

Detector	Apollo	Apollo	Apollo
Frame rate (Hz)	2	0.3	2
			
**Data collection and processing**			
Stage rotation rate (°/s)	2	0.3	2
Data collection time (s)	50	333	50
Electron flux density (e^−^/Å^2^/s)	0.01	0.01	0.045
Total fluence (e^−^/Å^2^)	0.5	3.33	2.25
Resolution (Å)	20–0.8 (0.9–0.8)	20–0.8 (0.9–0.8)	20–0.8 (0.9–0.8)
Space group	*P*2_1_2_1_2_1_	*P*2_1_2_1_2_1_	*P*2_1_2_1_2_1_
*a*, *b*, *c* (Å)	6.62, 17.84, 27.37	6.66, 18.12, 27.51	6.64, 18.06, 27.33
α, β, γ (°)	90,90,90	90,90,90	90,90,90
# total reflections	13477 (3656)	13789 (3838)	13550 (3818)
# unique reflections	2788 (801)	3215 (928)	3079 (879)
*R*_merge_ (%)	15.40 (34.70)	10.70 (149.50)	14.50 (83.00)
CC1/2 (%)	99.1 (47.9)	99.5 (33.2)	99.1 (51.5)
<*I*/σ*I*>	6.08 (3.97)	5.34 (0.74)	6.02 (1.46)
Completeness (%)	74.0 (74.1)	82.9 (82.9)	80.5 (80.6)
			
**Phasing**			
N trials	50000	50000	50000
N trials with CFOM > 80	2310	3810	3451
			
**Refinement**			
Resolution (Å)	20–0.8 (0.9–0.8)	20–0.8 (0.9–0.8)	20–0.8 (0.9–0.8)
*R*_1_/*R*1_all_ (%)	20.82/24.32	11.67/17.07	11.75/15.51
*wR*2 (%)	51.75	36.41	36.46
GooF	1.684	1.053	1.150
# atoms placed by *SHELXD*	40	40	40
# H-atoms seen in *F*_o_–*F*_c_ map	4	2	0

**Table 2:** Structures from biotin single crystals

Detector	Apollo	Apollo	Apollo
Frame rate (Hz)	2	0.3	2
			
**Data collection and processing**			
Stage rotation rate (°/s)	2	0.3	2
Data collection time (s)	50	333	50
Electron flux density (e^−^/Å^2^/s)	0.01	0.01	0.045
Total fluence (e^−^/Å^2^)	0.5	3.33	2.25
Resolution (Å)	20–0.8 (0.9–0.8)	20–0.8 (0.9–0.8)	20–0.8 (0.9–0.8)
Space group	*P*2_1_2_1_2_1_	*P*2_1_2_1_2_1_	*P*2_1_2_1_2_1_
*a*, *b*, *c* (Å)	5.12, 10.15, 20.56	5.11, 10.18, 20.76	5.09, 10.08, 20.65
α, β, γ (°)	90,90,90	90,90,90	90,90,90
# total reflections	4456 (1220)	4422 (1184)	2840 (782)
# unique reflections	1265 (359)	1126 (324)	1024 (367)
*R*_merge_ (%)	15.3 (26.9)	14.6 (45.4)	11.9 (28.2)
CC1/2 (%)	97.5 (67.5)	99.0 (77.5)	97.8 (86.3)
<*I*/σ*I*>	5.92 (4.25)	5.66 (2.53)	5.55 (2.83)
Completeness (%)	95.0 (95.7)	85.3 (86.9)	79.0 (80.1)
			
**Phasing**			
Best *SHELXT* CFOM	0.5389	0.6603	0.7221
N trials (*SHELXT*)	6400	6400	6400
N trials (*SHELXD*)	50000	50000	50000
N trials with CFOM > 80 (*SHELXD*)	7616	7693	265
			
**Refinement**			
Resolution (Å)	20–0.8 (0.9–0.8)	20–0.8 (0.9–0.8)	20–0.8 (0.9–0.8)
*R*_1_/*R*1_all_ (%)	17.88/22.43	15.86/18.56	19.42/21.05
w*R*_2_ (%)	48.78	40.94	50.02
GooF	1.626	1.325	1.652
# atoms placed by *SHELXT*	16	16	16
# H-atoms seen in *F*_o_–*F*_c_ map	2	1	4

**Table 3:** Structures from thio­strepton single crystals

Detector	Apollo	Apollo	Apollo
Frame rate (Hz)	2	0.3	2
			
**Data collection and processing**			
Stage rotation rate (°/s)	2	0.3	2
Data collection time (s)	50	333	50
Electron flux density (e^−^/Å^2^/s)	0.01	0.01	0.045
Total fluence (e^−^/Å^2^)	0.5	3.33	2.25
Resolution (Å)	19.09–2.0 (2.1–2.0)	18.72–1.5 (1.6–1.5)	15.56–1.81 (1.9–1.81)
Space group	*P*4_3_2_1_2	*P*4_3_2_1_2	*P*4_3_2_1_2
*a*, *b*, *c* (Å)	27.00, 27.00, 27.51	26.47, 26.47, 27.29	26.68, 26.68. 27.52
α, β, γ (°)	90,90,90	90,90,90	90,90,90
# total reflections	4938	12605 (2143)	5437 (761)
# unique reflections	717 (88)	1738 (289)	969 (129)
*R*_merge_ (%)	27.0 (82.4)	20.4 (117.8)	24.7 (66.6)
CC1/2 (%)	98.6 (67.1)	98.5 (57.2)	98.3 (60.1)
<*I*/σ*I*>	5.41 (2.61)	6.67 (1.89)	5.46 (2.51)
Completeness (%)	88.2 (83.0)	99.3 (98.6)	91.2 (88.4)
			
**Phasing**			
Search model PDB	1e9w	1e9w	1e9w
			
**Refinement**			
Resolution (Å)	19.09–2.0	18.72–1.50	15.56–1.81
*R*_work_ (%)	21.89	20.26	23.37
*R*_free_ (%)	29.39	21.67	30.19
# protein atoms	118	118	118
# solvent mol­ecules	1	2	1
Average *B*factor	11.07	13.09	13.83
