# Supplementary material for: Fast event-based electron counting for small-mol­ecule structure determination by MicroED
Source: Acta Crystallogr C Struct Chem. 2025 Feb 21;81(Pt 3):116–30. doi: 10.1107/S2053229624012300 (PMC11881165; doi:10.1107/S2053229624012300)
Supplement: Supplementary file 2 [file c-81-00116-sup2.pdf]

Acta Cryst C
STRUCTURAL CHEMISTRY

Volume 81 (2025)

Supporting information for article:

Fast event-based electron counting for small-molecule structure determination by MicroED

Niko Vlahakis, Songrong Qu, Logan S. Richards, Lygia Silva de Moraes, Duilio Cascio, Hosea M. Nelson and Jose A. Rodriguez

Supplementary script 1. Calculation of CL estimates for a given incident electron flux per pixel per second. This script requires an explicit count rate and conversion of counts to electrons. It is configured to run in Matlab version 2023b, in a parallelized manner, with access to 16 cores.

```
function[countsout,lostcounts]=ebecsim()
%Required:
%anticipated detector count rate (per pix per sec)
%flux range (per pix per sec)
%ntrials = number of measurements simulated

%% COUNTING SIMULATION

countrate=2392; %internal detector count rate (e-/pix/s)
fluxin=500; %incident beam flux (e-/pix/s)
cpix=9; %number of correlated pixels used during counting and centroiding
ntrials=1000; %number of independent measurements simulated

countsout = ones(fluxin,ntrials,'single');
lostcounts = zeros(fluxin,ntrials,'single');

for aa=2:fluxin
    for bb=1:ntrials
        rng("shuffle");
        diste=randi([1 countrate],1,cpix.*aa);
        grosscounts=hist(diste,1:countrate);
        netcounts=grosscounts;
        netcounts(netcounts>0)=1;
        ncounts=sum(netcounts)./cpix;
        countsout(aa,bb)=ncounts;
        lcounts=(sum(grosscounts)./cpix)-ncounts;
        lostcounts(aa,bb)=lcounts;
    end
end
```

Supplementary script 2. SerialEM script for EBEC data collection of continuous rotation MicroED data on the Apollo detector. The script assumes crystal locations have been saved in the navigator and recalls those locations for a set number of items. It also allows for multiple sweeps to be recorded from a single crystal. Lastly, the script assumes the Apollo detector is inserted and gain normalized, and that it is configured, within the SerialEM environment to record exposures of the desired time/degree length.

```
ScriptName BatchED

SetColumnOrGunValve 1
SetBeamBlank 0

nitems = 5
xtalct = 1
Startangle = 50
Finalangle = -50
nsweeps = 1
rotrate = 0.0666

Loop $nitems
   xtalct = $xtalct + 1
   sweepcount = 1
   SetFolderForFrames mov$xtalct_sweep$sweepcount
   MoveToNavItem $xtalct

   Loop $nsweeps
      sweepcount = $sweepcount + 1
      TiltTo $Startangle
      Delay 2 sec
      GoToLowDoseArea S
      UseContinuousFrames 1

      echo STARTING TILT
      BackgroundTilt $Finalangle $rotrate
      Delay 1 sec
      ReportStageXYZ
      Search
      StopContinuous

      echo END TILT
      ReportClock
      ReportStageXYZ
      Delay 20 sec
   EndLoop

EndLoop
```

Supplementary script 3. Adjustment of electron counts per pixel per second in a given dataset, to account for anticipated CL as calculated in Supplementary script 1. The raw, super-resolution MRC file written by the Apollo, the pre-calculated simulations of CL generated by Supplementary Script 1, and an output name are required as inputs to this script, The CL-adjustment script is configured to run in Matlab version 2023b; the code can be executed in parallel, with access to sufficient memory resources.

```
function[diffstackout,cl_residuals]=mrc_clfix(diffstackin,ebecsimdata)
% Required:
%diffstackin: diffraction data matrix
%ebecsimdata: data file containing simulated CL for a given detector

cmin=10;
cmax=266;
ctstoelectrons=16;
fps=2;

load(ebecsimdata,'countsout','lostcounts');
ctsmean=mean(countsout,2);
clmean=mean(lostcounts,2);

diffstackout=diffstackin;
for jj=cmin:cmax
    tmpstack=abs(diffstackin-jj);
    tmpstack(tmpstack>2)=0;
    tmpstack(tmpstack>0)=1;
    pixpool=sum(sum(sum(tmpstack)));
    if pixpool>1
        [~,fluxin]=min(abs(ctsmean-jj));
        closs=clmean(fluxin);
        diffstackout(tmpstack==1)=diffstackout(tmpstack==1) + closs;
    end
end

cl_residuals=diffstackout-diffstackin;
```

Supplementary Table 1 Electron counts as a function of incident beam flux in static MicroED data collected from salen ligand crystals on the Apollo detector.

**Condition**	**Maximum Counts (e^-/pix/s)**			**Maximum percentage of signal pixels with counts > 80 e^-/pix/s per frame**		
Spot size	9	10	11	9	10	11
Incident flux (e^-/Å^2/s)	0.045	0.03	0.01	0.045	0.03	0.01
Crystal 1	203	196	246	5.3	5.2	4.5
Crystal 2	114	103	49	3.1	2.8	1.7
Crystal 3	107	13	8	2.1	1.6	1.2
Crystal 4	-	107	87	-	2.4	1.9
Crystal 5	131	91	53	3.2	2.7	2.6
Crystal 6	71	60	26	3.3	2.5	2.9

Supplementary Table 2 Difference map peaks greater than 3σ in EBEC structures of thiostrepton determined at different incident fluence, when the data is refined against a model of thiostrepton mutated to poly-alanine

Total incident fluence ($e^-/Å^2$)	Residue/sidechain/heteroatom	Sigma level of peak
**0.50**	Residue 6 thiazole	+4.04 RMSD
	Water	+3.72 RMSD
	Residue 7 threonine sidechain	+3.48 RMSD
	Residue 11 thiazole	+3.40 RMSD
	Residue 3 ethylene	+3.34 RMSD
**3.33**	Residue 11 thiazole	+5.81 RMSD
	Residue 13 thiazole	+5.04 RMSD
	Residue 6 thiazole	+4.60 RMSD
	Residue 8 dehydrobutyrine sidechain	+4.08 RMSD
	Residue 9 thiazole	+3.90 RMSD
	Residue 16 ethylene	+3.32 RMSD
	Residue 12 threonine sidechain	+3.14 RMSD
	Residue 15 thiazole	+3.02 RMSD
**2.25**	Residue 11 thiazole	+5.70 RMSD
	Residue 8 dehydrobutyrine sidechain	+4.37 RMSD
	Residue 6 thiazole	+4.28 RMSD
	Residue 9 thiazole	+4.10 RMSD
	Residue 15 thiazole	+3.39 RMSD
	Residue 13 thiazole	+3.31 RMSD
	Water	+3.01 RMSD

Supplementary Table 3 Data reduction statistics for the representative salen ligand EBEC datasets featured in Table 1 considering only commonly observed Miller indices across all three experiments

**Detector**	Apollo	Apollo	Apollo
Frame rate (Hz)	2	0.3	2
**Data Collection and Processing**			
Stage rotation rate (°/s)	2	0.3	2
Data collection time (s)	50	333	50
Electron Flux (e^-/Å^2/s)	0.01	0.01	0.045
Total Fluence (e^-/Å^2)	0.5	3.33	2.25
Resolution (Å)	20 – 0.8 (0.9-0.8)	20 – 0.8 (0.9-0.8)	20 – 0.8 (0.9-0.8)
Space Group	$P2_12_12_1$	$P2_12_12_1$	$P2_12_12_1$
a, *b*, *c* (Å)	6.62, 17.84, 27.37	6.66, 18.12, 27.51	6.64, 18.06, 27.33
α, β, γ (°)	90,90,90	90,90,90	90,90,90
# total reflections	5666 (1537)	5577 (1425)	5595 (1468)
# unique reflections	1842 (511)	1845 (478)	1858 (494)
R_{merge} (%)	15.40 (34.70)	9.4 (124.2)	12.5 (69.5)
CC1/2 (%)	98.9 (37.6)	99.7 (18.2)	99.0 (41.5)
<I/σI>	4.65 (3.44)	4.68 (0.70)	5.48 (1.37)
Completeness (%)	48.9 (47.3)	47.6 (42.7)	48.6 (45.3)

Supplementary Table 4 Refinement statistics for the representative biotin EBEC datasets featured in Table 2 considering only commonly observed Miller indices across all three experiments

**Detector**	Apollo	Apollo	Apollo
Frame rate (Hz)	2	0.3	2
**Data Collection and Processing**			
Stage rotation rate (°/s)	2	0.3	2
Data collection time (s)	50	333	50
Electron Flux ($e^-/Å^2/s$)	0.01	0.01	0.045
Total Fluence ($e^-/Å^2$)	0.5	3.33	2.25
Resolution (Å)	20 – 0.8 (0.9-0.8)	20 – 0.8 (0.9-0.8)	20 – 0.8 (0.9-0.8)
Space Group	$P2_12_12_1$	$P2_12_12_1$	$P2_12_12_1$
a, *b*, *c* (Å)	5.12, 10.15, 20.56	5.11, 10.18, 20.76	5.09, 10.08, 20.65
α, β, γ (°)	90,90,90	90,90,90	90,90,90
# total reflections	1021 (263)	1026 (261)	1018 (275)
# unique reflections	333 (95)	339 (94)	336 (98)
R_{merge} (%)	13.5 (30.7)	11.4 (27.6)	15.4 (39.0)
CC1/2 (%)	98.0 (44.7)	99.3 (78.4)	97.5 (75.5)
<I/σI>	5.61 (3.46)	5.90 (2.77)	5.00 (2.41)
Completeness (%)	25.0 (25.3)	25.7 (25.2)	25.9 (26.7)

Supplementary Table 5 Statistics for the representative thiostrepton EBEC datasets featured in Table 3 following rigid-body refinement of reflection files only including miller indices commonly observed across all three trials, with the same set of R-free flags for each, against the 3.33 $e^-/Å^2$ fluence model refined for deposition (PDB: 9CQ0).

**Detector**	Apollo	Apollo	Apollo
Frame rate (Hz)	2	0.3	2
Stage rotation rate (°/s)	2	0.3	2
Data collection time (s)	50	333	50
Electron Flux ($e^-/Å^2/s$)	0.01	0.01	0.045
Total Fluence ($e^-/Å^2$)	0.5	3.33	2.25
Resolution (Å)	12.26 – 2.0 (2.1-2.0)	12.13 – 2.0 (2.1 – 2.0)	12.23 – 2.0 (2.1 -2.0)
Space Group	$P4_32_12$	$P4_32_12$	$P4_32_12$
a, *b*, *c* (Å)	27.00, 27.00, 27.51	26.47, 26.47, 27.30	26.68, 26.68. 27.52
α, *β*, *γ* (°)	90,90,90	90,90,90	90,90,90
# unique reflections	663	664	665
Completeness (%)	86.44	86.91	87.04
R_{work} (%)	30.57	18.43	25.48
R_{free} (%)	34.49	21.85	29.27
# protein atoms	118	118	118
# solvent molecules	2	2	2
Average B-factor	13.09	13.09	13.09

Supplementary Table 6 Difference map peaks greater than 3σ in EBEC structures of thiostrepton determined at different incident fluence, with only reflections of commonly observed Miller index across all three experiments used in refinement and the same R-free test set of reflections conserved across all trials. For this assessment, the data is refined against a model of thiostrepton mutated to poly-alanine.

Total incident fluence ($e^-/Å^2$)	Residue/sidechain/heteroatom	Sigma level of peak
**0.50**	Residue 11 thiazole	+4.07 RMSD
	Residue 8 dehydrobutyrine sidechain	+3.58 RMSD
	Residue 7 threonine sidechain	+3.53 RMSD
	Residue 6 thiazole	+3.49 RMSD
	Residue 13 thiazole	+3.42 RMSD
	Spurious water-like peak	+3.40 RMSD
	Residue 3 ethylene	+3.31 RMSD
**3.33**	Residue 11 thiazole	+4.95 RMSD
	Residue 6 thiazole	+4.49 RMSD
	Residue 8 dehydrobutyrine sidechain	+4.01 RMSD
	Residue 15 thiazole	+3.89 RMSD
	Residue 13 thiazole	+3.51 RMSD
	Residue 9 thiazole	+3.36 RMSD
**2.25**	Residue 11 thiazole	+4.63 RMSD
	Residue 8 dehydrobutyrine sidechain	+4.13 RMSD
	Residue 7 threonine	+3.68 RMSD
	Residue 15 thiazole	+3.58 RMSD
	Residue 6 thiazole	+3.10 RMSD
	Residue 9 thiazole	+3.04 RMSD
	Residue 13 thiazole	+3.03 RMSD

Supplementary Table 7 Twin law test results (Reported as <L> ; <L^2> ; **Estimated twin fraction**) of unmodified and CL-adjusted EBEC MicroED datasets.

Spot size	9	9	11	11	11	11
Flux (e$^-$ / Å^2 /s)	0.045	0.045	0.01	0.01	0.01	0.01
Rotation rate (deg/s)	2	2	2	2	0.3	0.3
CL-adjusted	-	+	-	+	-	+
**Salen ligand**						
Crystal 1	0.490; 0.321; **0**	0.490; 0.313; **0.011**	0.433; 0.261; **0.119**	0.466; 0.294; **0.047**	0.505; 0.339; **0**	0.477; 0.308; **0.030**
Crystal 2	0.481; 0.321; **0.011**	0.483; 0.313; **0.020**	0.460; 0.287; **0.057**	0.476; 0.305; **0.031**	0.496; 0.329; **0**	0.480; 0.311; **0.025**
Crystal 3	0.482; 0.310; **0.024**	0.483; 0.313; **0.021**	0.441; 0.267; **0.097**	0.473; 0.301; **0.036**	0.507; 0.340; **0**	0.463; 0.292; **0.052**
Crystal 4	0.489; 0.318; **0.013**	0.492; 0.321; **0.009**	0.493; 0.323; **0.007**	0.493; 0.324; **0.007**	0.515; 0.350; **0**	0.502; 0.335; **0**
Crystal 5	0.482; 0.313; **0.023**	0.477; 0.307; **0.028**	0.481; 0.314; **0.023**	0.480; 0.311; **0.024**	0.519; 0.357; **0**	0.510; 0.347; **0**
**Biotin**						
Crystal 1	0.516; 0.350; **0**	0.518; 0.352; **0**	0.439; 0.266; **0.102**	0.474; 0.301; **0.034**	0.510; 0.342; **0**	0.466; 0.294; **0.047**
Crystal 2	0.495; 0.325; **0.006**	0.495; 0.325; **0.005**	0.448; 0.273; **0.083**	0.482; 0.311; **0.022**	0.547; 0.386; **0**	0.501; 0.334; **0**
Crystal 3	0.525; 0.359; **0**	0.533; 0.368; **0**	0.432; 0.258; **0.121**	0.448; 0.274; **0.082**	0.528; 0.364; **0**	0.502; 0.333; **0**
Crystal 4	0.477; 0.304; **0.029**	0.479; 0.305; **0.026**	0.428; 0.260; **0.132**	0.498; 0.330; **0**	0.506; 0.335; **0**	0.466; 0.294; **0.046**
Crystal 5	0.500; 0.331; **0**	0.498; 0.329; **0**	0.469; 0.296; **0.042**	0.505; 0.335; **0**	0.511; 0.344; **0**	0.490; 0.320; **0.012**
Crystal 6	N/A	N/A	0.440; 0.269; **0.101**	0.496; 0.327; **0**	N/A	N/A
**Thiostrepton**						
Crystal 1	0.450; 0.278; **0.077**	N/A	0.466; 0.297; **0.048**	N/A	0.419; 0.247; **0.159**	N/A

Crystal 2	0.463; 0.293; **0.052**	N/A	0.445; 0.273; **0.081**	N/A	0.469; 0.299; **0.042**	N/A
Crystal 3	0.447; 0.277; **0.083**	N/A	0.454; 0.283; **0.069**	N/A	0.465; 0.295; **0.048**	N/A
Crystal 4	0.463; 0.293; **0.053**	N/A	0.481; 0.315; **0.023**	N/A	0.478; 0.307; **0.028**	N/A
Crystal 5	0.458; 0.288; **0.061**	N/A	N/A	N/A	N/A	N/A
Crystal 6	0.474; 0.303; **0.034**	N/A	N/A	N/A	N/A	N/A

Supplementary Table 8 Crystallographic data reduction statistics for MicroED datasets from individual crystals of the salen ligand collected first on the Ceta-D detector, and then on the Apollo detector, with an incident flux of 0.01 $e^-/Å^2/s$, and equivalent rotation and sampling rates.

**Crystal**	**1**	**1**	**2**	**2**
Electron Flux (e^- / $Å^2$ /s)	0.01	0.01	0.01	0.01
Total Fluence (e^- / $Å^2$)	0.5	0.5	0.5	0.5
Stage rotation rate (°/s)	2	2	2	2
Data collection time (s)	50	50	50	50
**Detector**	Apollo	Ceta-D	Apollo	Ceta-D
Frame rate (Hz)	2	2	2	2
**Data Processing**				
Resolution (Å)	20 – 0.8 (0.9-0.8)	20 – 0.8 (0.9-0.8)	20 – 0.8 (0.9-0.8)	20 – 0.8 (0.9-0.8)
# total reflections	13394 (3671)	13682 (3175)	13328 (3706)	13404 (3029)
# unique reflections	3469 (989)	3539 (842)	2670 (777)	2670 (622)
R_{merge} (%)	22.10 (38.20)	54.20 (1344.20)	25.40 (90.00)	63.80 (9624.80)
CC1/2 (%)	97.8 (36.8)	94.0 (-7.5)	97.7 (24.3)	93.2 (-4.2)
<I/σI>	4.35 (3.36)	1.42 (0.09)	4.05 (1.53)	1.62 (0.00)
Completeness (%)	92.40 (91.90)	88.20 (72.80)	71.60 (72.30)	66.70 (53.90)
**Crystal**	**3**	**3**	**4**	**4**
Electron Flux (e^- / $Å^2$ /s)	0.01	0.01	0.01	0.01
Total Fluence (e^- / $Å^2$)	0.5	0.5	0.5	0.5
Stage rotation rate (°/s)	2	2	2	2
Data collection time (s)	50	50	50	50
**Detector**	Apollo	Ceta-D	Apollo	Ceta-D
Frame rate (Hz)	2	2	2	2
**Data Processing**				
Resolution (Å)	20 – 0.8 (0.9-0.8)	20 – 0.8 (0.9-0.8)	20 – 0.8 (0.9-0.8)	20 – 0.8 (0.9-0.8)
# total reflections	13408 (3780)	13639 (3058)	13351 (3675)	12507 (3215)
# unique reflections	3676 (1057)	3651 (830)	2065 (606)	2068 (538)
R_{merge} (%)	21.00 (83.90)	55.90 (980.40)	16.30 (21.60)	48.00 (716.70)
CC1/2 (%)	98.3 (33.6)	95.6 (2.4)	98.9 (59.3)	97.5 (-12.9)
<I/σI>	3.85 (1.20)	1.59 (0.10)	7.40 (6.54)	1.77 (1.77)
Completeness (%)	98.30 (98.90)	90.40 (71.70)	55.70 (57.0)	51.80 (46.90)
**Crystal**	**5**	**5**		

Electron Flux (e^- / $Å^2$ /s)	0.01	0.01
Total Fluence (e^- / $Å^2$)	0.5	0.5
Stage rotation rate (°/s)	2	2
Data collection time (s)	50	50
**Detector**	Apollo	Ceta-D
Frame rate (Hz)	2	2
**Data Processing**		
Resolution (Å)	20 – 0.8 (0.9-0.8)	20 – 0.8 (0.9-0.8)
# total reflections	12426 (3752)	14397 (3846)
# unique reflections	3633 (1046)	3825 (1006)
R_{merge} (%)	22.10 (78.60)	56.30 (-99.90)
CC1/2 (%)	97.7 (19.7)	93.9 (-14.0)
<I/σI>	3.75 (1.47)	1.43 (0.00)
Completeness (%)	97.40 (97.80)	94.90 (86.50)

Supplementary Table 9 Crystallographic data reduction statistics for MicroED datasets from individual crystals of the salen ligand collected first on the Ceta-D detector, and then on the Apollo detector, with an incident flux of 0.045 e$^-$/Å^2/s, and equivalent rotation and sampling rates.

**Crystal**	**1**	**1**	**2**	**2**
Electron Flux (e$^-$ / Å^2 /s)	0.045	0.045	0.045	0.045
Total Fluence (e$^-$ / Å^2)	2.25	2.25	2.25	2.25
Stage rotation rate (°/s)	2	2	2	2
Data collection time (s)	50	50	50	50
**Detector**	Apollo	Ceta-D	Apollo	Ceta-D
Frame rate (Hz)	2	2	2	2
**Data Processing**				
Resolution (Å)	20 – 0.8 (0.9-0.8)	20 – 0.8 (0.9-0.8)	20 – 0.8 (0.9-0.8)	20 – 0.8 (0.9-0.8)
# total reflections	9800 (2693)	14261 (3959)	12898 (3530)	14057 (3628)
# unique reflections	2556 (728)	2703 (750)	3409 (950)	3505 (888)
R_{merge} (%)	19.00 (253.30)	30.50 (896.90)	20.10 (77.70)	64.90 (-99.90)
CC1/2 (%)	98.7 (2.8)	98.5 (-3.5)	98.3 (31.8)	93.7 (-5.5)
<I/σI>	3.23 (0.48)	3.14 (0.18)	4.3 (1.52)	1.33 (0.00)
Completeness (%)	68.30 (67.50)	67.50 (65.30)	90.70 (88.70)	86.40 (75.60)
**Crystal**	**3**	**3**	**4**	**4**
Electron Flux (e$^-$ / Å^2 /s)	0.045	0.045	0.045	0.045
Total Fluence (e$^-$ / Å^2)	2.25	2.25	2.25	2.25
Stage rotation rate (°/s)	2	2	2	2
Data collection time (s)	50	50	50	50
**Detector**	Apollo	Ceta-D	Apollo	Ceta-D
Frame rate (Hz)	2	2	2	2
**Data Processing**				
Resolution (Å)	20 – 0.8 (0.9-0.8)	20 – 0.8 (0.9-0.8)	20 – 0.8 (0.9-0.8)	20 – 0.8 (0.9-0.8)
# total reflections	13161 (3655)	14458 (4048)	13210 (3665)	14410 (4037)
# unique reflections	2572 (730)	2830 (802)	2262 (650)	2358 (669)
R_{merge} (%)	13.80 (99.80)	21.10 (227.20)	15.30 (100.50)	20.50 (216.70)
CC1/2 (%)	99.3 (29.6)	98.9 (2.5)	99.0 (46.4)	98.5 (23.0)
<I/σI>	5.99 (1.35)	3.9 (0.57)	5.92 (1.39)	4.44 (0.72)
Completeness (%)	68.60 (67.80)	70.80 (69.90)	60.40 (60.40)	58.90 (58.30)
**Crystal**	**5**	**5**		

Electron Flux (e^- / $Å^2$ /s)	0.045	0.045
Total Fluence (e^- / $Å^2$)	2.25	2.25
Stage rotation rate (°/s)	2	2
Data collection time (s)	50	50
**Detector**	Apollo	Ceta-D
Frame rate (Hz)	2	2
**Data Processing**		
Resolution (Å)	20 – 0.8 (0.9-0.8)	20 – 0.8 (0.9-0.8)
# total reflections	13352 (3699)	14566 (4085)
# unique reflections	2719 (773)	2886 (827)
R_{merge} (%)	22.40 (378.00)	26.20 (307.20)
CC1/2 (%)	99.1 (0.3)	97.9 (9.1)
<I/σI>	3.63 (0.37)	3.20 (0.44)
Completeness (%)	72.60 (71.80)	71.90 (71.70)

Supplementary Table 10 List of Zenodo archive entries where diffraction data from this report is accessible.

**Dataset**	**Zenodo DOI**
EBEC diffraction tilt series on biotin nanocrystals at variable total fluence	https://zenodo.org/doi/10.5281/zenodo.13690162
EBEC diffraction tilt series on salen ligand nanocrystals at variable total fluence	https://zenodo.org/doi/10.5281/zenodo.13713098
EBEC diffraction tilt series on thiostrepton nanocrystals at variable total fluence	https://zenodo.org/doi/10.5281/zenodo.13716076
EBEC diffraction movies over a range of incident flux on salen ligand crystals	https://zenodo.org/doi/10.5281/zenodo.13716389
EBEC diffraction movies over a range of incident flux on salen ligand crystals	https://zenodo.org/doi/10.5281/zenodo.13716389
Diffraction tilt series recorded on the DE Apollo and Ceta D camera for the same salen ligand crystals, at variable incident flux	https://zenodo.org/doi/10.5281/zenodo.13716389

Supplementary figures.

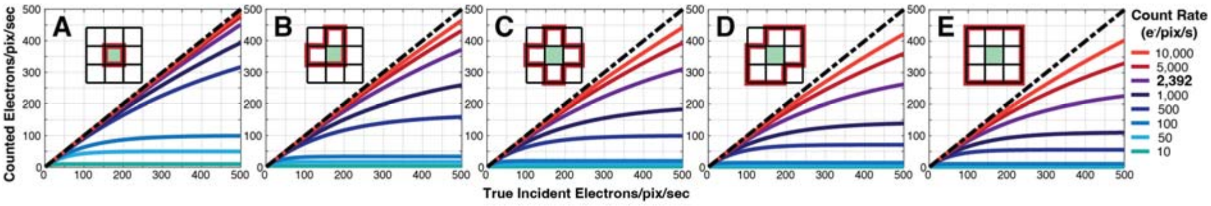

Supplementary figure 1. Simulated electron counts from different clusters of pixels, sampled on a one second interval with a given count rate. Sampled count rates: 10, 50, 100, 500, 1000, 2392, 5000 and 10000 e^-/pix/s. Curves show the effect of the varied internal pixel count rates on the expected total count of electrons per pixel for a given true electron flux on that pixel assuming no correlation with its neighbors (A), and a progressively larger cluster of pixels considered during counting: 3, 5, 7, and 9 (B-E). A dashed black line denotes the hypothetical line corresponding to perfect counting. The maximum internal count rate for the Apollo detector is denoted in bold: 2,392 e^-/pix/s.

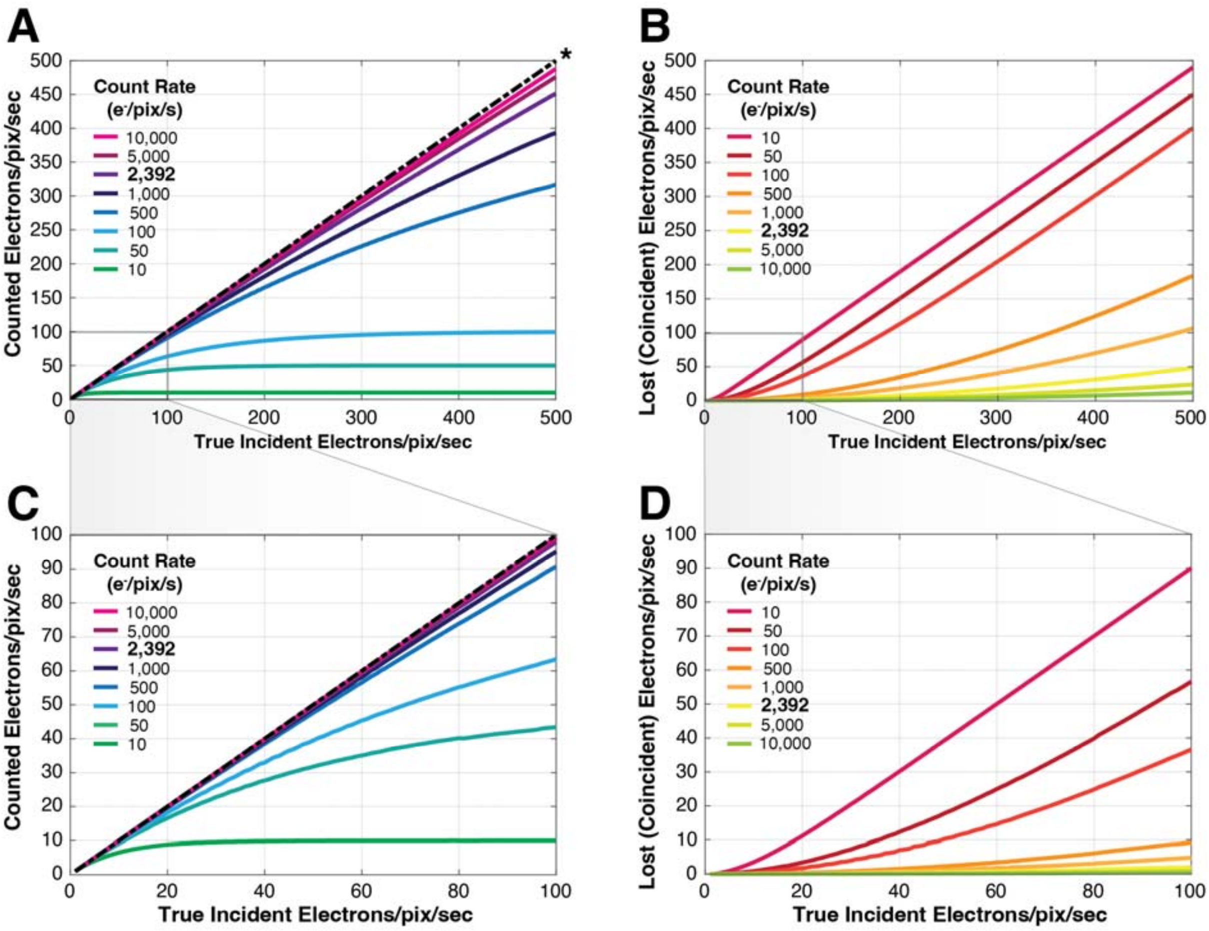

Supplementary figure 2. Simulated electron counts from a single hypothetical pixel, sampled on a one second interval with a given count rate. Sampled count rates: 10, 50, 100, 500, 1000, 2392, 5000 and 10000 e$^-$/pix/s. Curves show the effect of the varied internal pixel count rates on the expected total count of electrons per pixel for a given true electron flux on that pixel (A). For each count rate, the expected count of electrons lost due to coincidence is shown as a function of incident electron flux on a pixel (B). Insets (C,D) magnify the region from 0 to 100 e$^-$/pix/s in (A,B). A star (A) denotes the hypothetical line corresponding to perfect counting. The maximum internal count rate for the Apollo detector is denoted in bold: 2,392 e$^-$/pix/s.

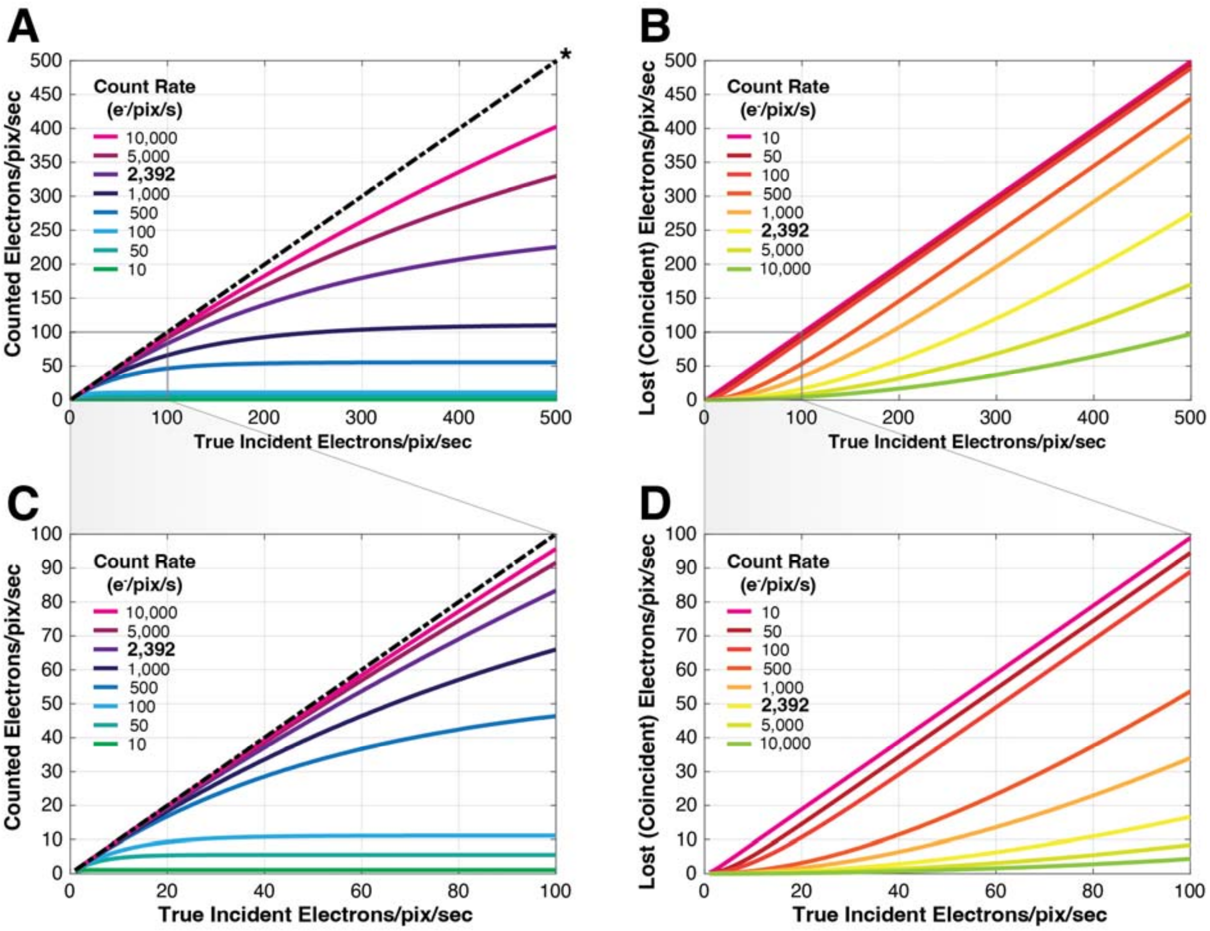

Supplementary figure 3. Simulated electron counts from a 3x3 cluster of hypothetical pixels, sampled on a one second interval with a given count rate per pixel. Sampled count rates: 10, 50, 100, 500, 1000, 2392, 5000 and 10000 e^-/pix/s. Curves show the effect of the varied internal pixel count rates on the expected total count of electrons per pixel for a given true electron flux on that pixel (A). For each count rate, the expected count of electrons lost due to coincidence is shown as a function of incident electron flux on a pixel (B). Insets (C,D) magnify the region from 0 to 100 e^-/pix/s in (A,B). A star (A) denotes the hypothetical line corresponding to perfect counting. The maximum internal count rate for the Apollo detector is denoted in bold: 2,392 e^-/pix/s.

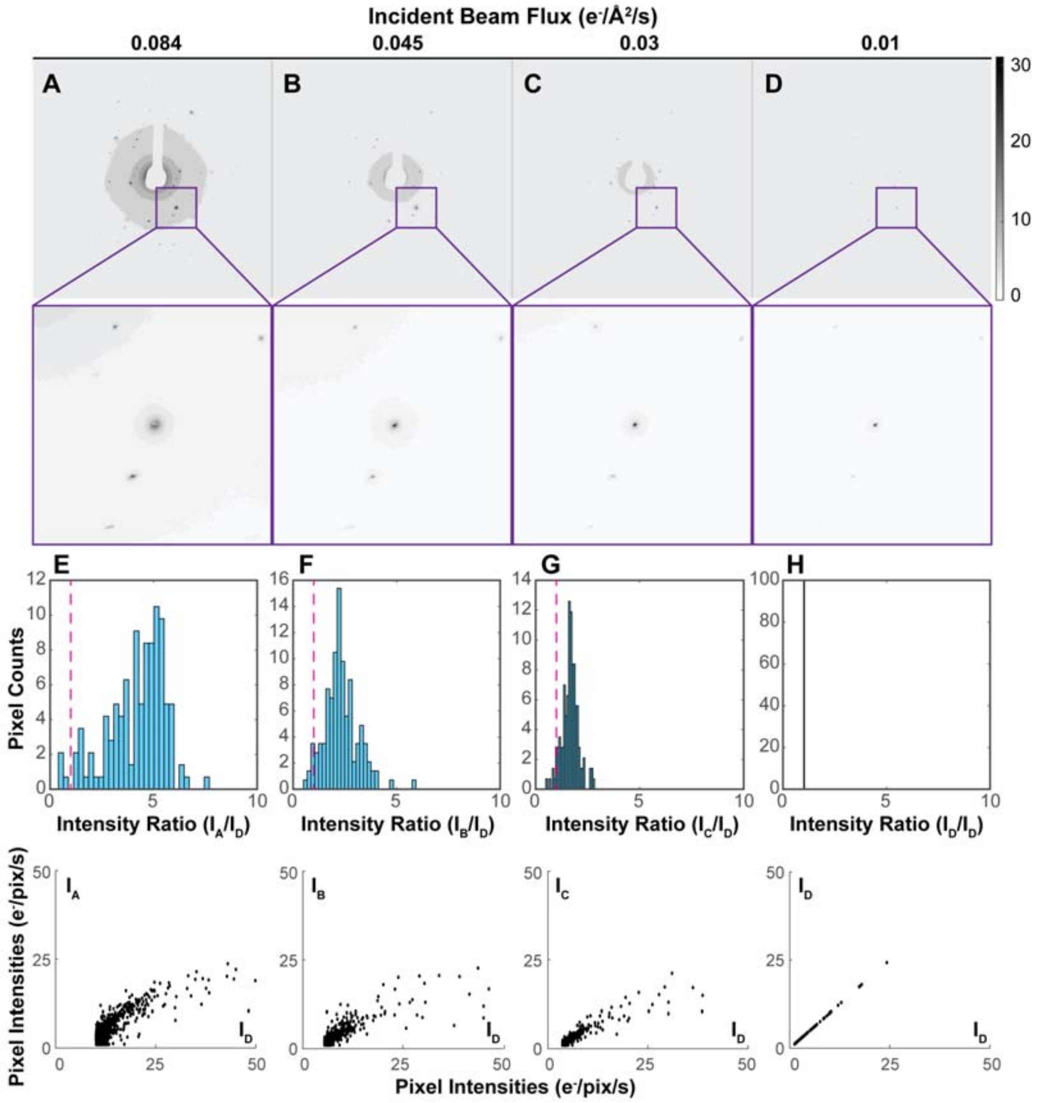

Supplementary figure 4. Measured diffraction from well ordered, highly diffracting nanocrystals of Co(II) meso-tetraphenyl porphyrin. (A-D) Diffraction obtained from the same illuminated region of the same crystal at increasing incident flux values, from 0.01 to 0.084 $e^-/Å^2/s$. Insets show a magnified view of reflections observed in outlined purple boxes, highlighting the strongest incident reflection. (E-H) Histograms of ratios of electrons counted in diffraction patterns shown in panels A-C, vs. panel D (top), where a magenta dashed line indicates a ratio of 1. All count ratios in panel H are 1, since values there are comparing panel D counts to themselves; all other count ratios are greater than zero. While most counts are greater than one in panels A-C, some counts are less than one, indicating loss of counts in higher incident flux patterns due to coincidence, or other count restrictions. Scatterplots (bottom) for each of E-H, compare electron counts for individual pixels in conditions A-C to those in D. Only pixels with values greater than 1 were considered in the analysis. Electron counts along the horizontal axes of these scatterplots are for flux values in D multiplied by 0.084 (E), 0.045 (F), and 0.03 (G)

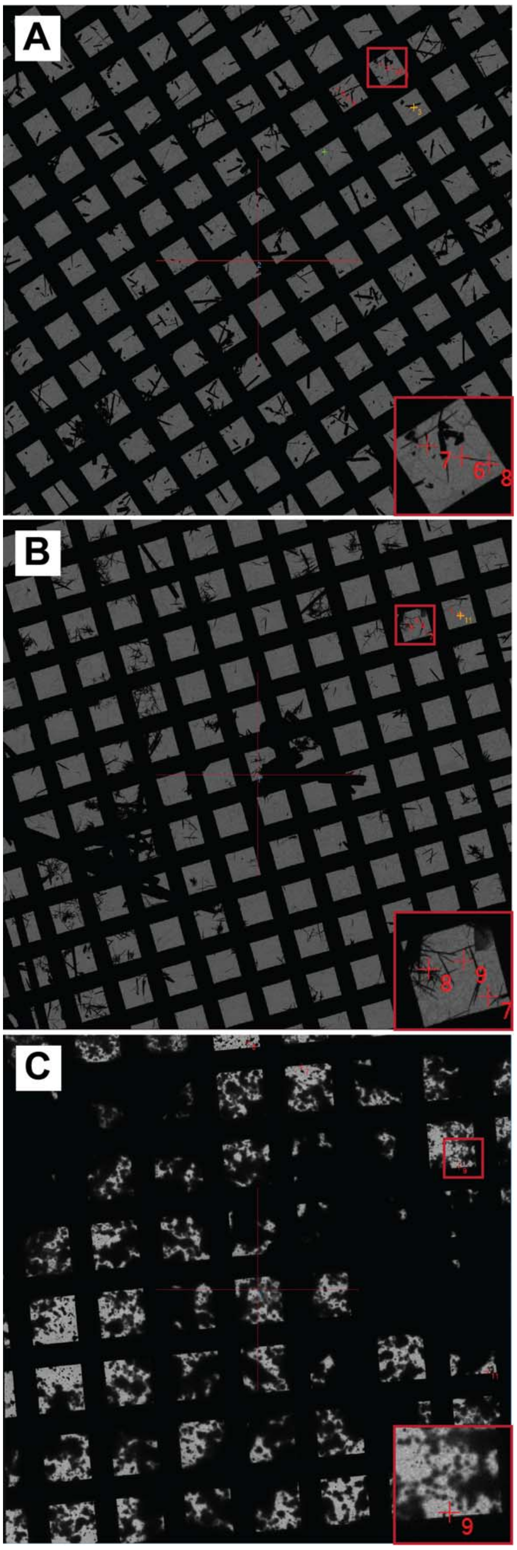

Supplementary figure 5. Overviews of grids containing salen ligand (A), biotin (B), and thiostrepton (C) crystals. Each overview is a montage of images pieced together by serialEM, and each is used to select crystals for fast EBEC MicroED data collection in automated fashion using serialEM. For each panel, red boxes outline a region of the grid that was targeted for data collection, and insets show the numbered locations of targeted crystals.

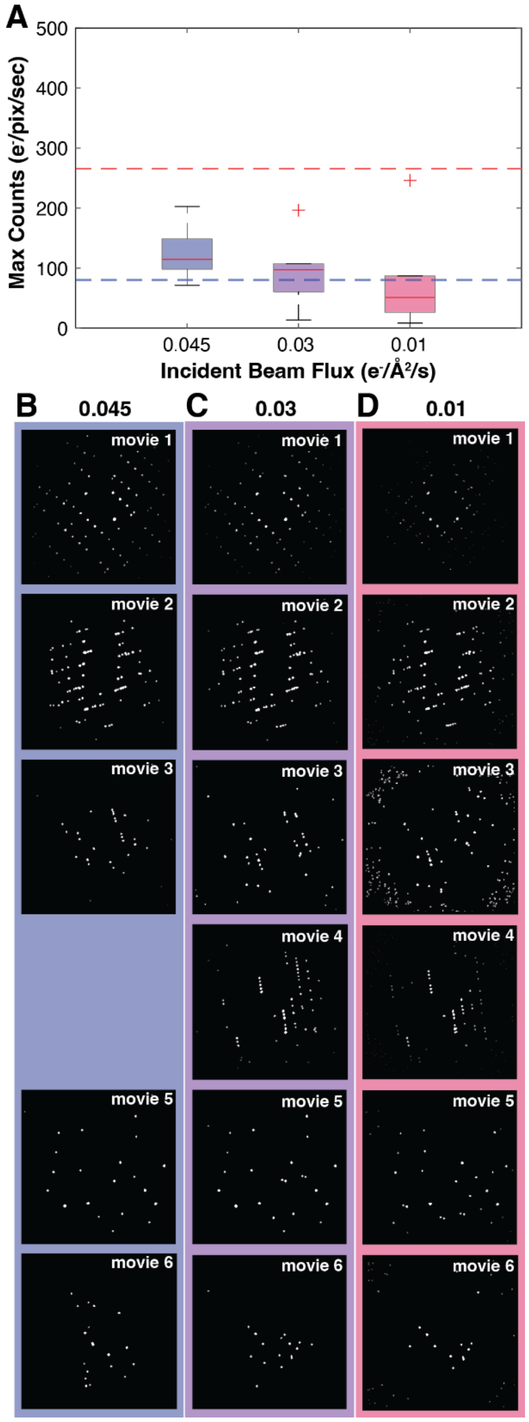

Supplementary figure 6. Analysis of diffraction recorded from salen ligand crystals illuminated with different incident beam flux. (A) The distribution of maximum counts recorded per pixel over a given dataset is shown for three incident beam flux conditions, 0.01, 0.03 and 0.045 e$^-$/Å^2/s; the dashed red line indicates 266 e$^-$/pix/s and dashed blue line, 80 e$^-$/pix/s. Each box charts the mean of and first and fourth quartiles of a maximum counts distribution; red crosses denote outliers. Maximum projections of all reflections detected in each dataset recorded for the different incident beam flux conditions are shown in (B-D). For numbered movie the same crystal was diffracted at the different incident beam flux values, yielding three congruent sets of patterns of varying diffraction intensity; movie 4 at the highest incident flux could not be processed due to a technical malfunction.

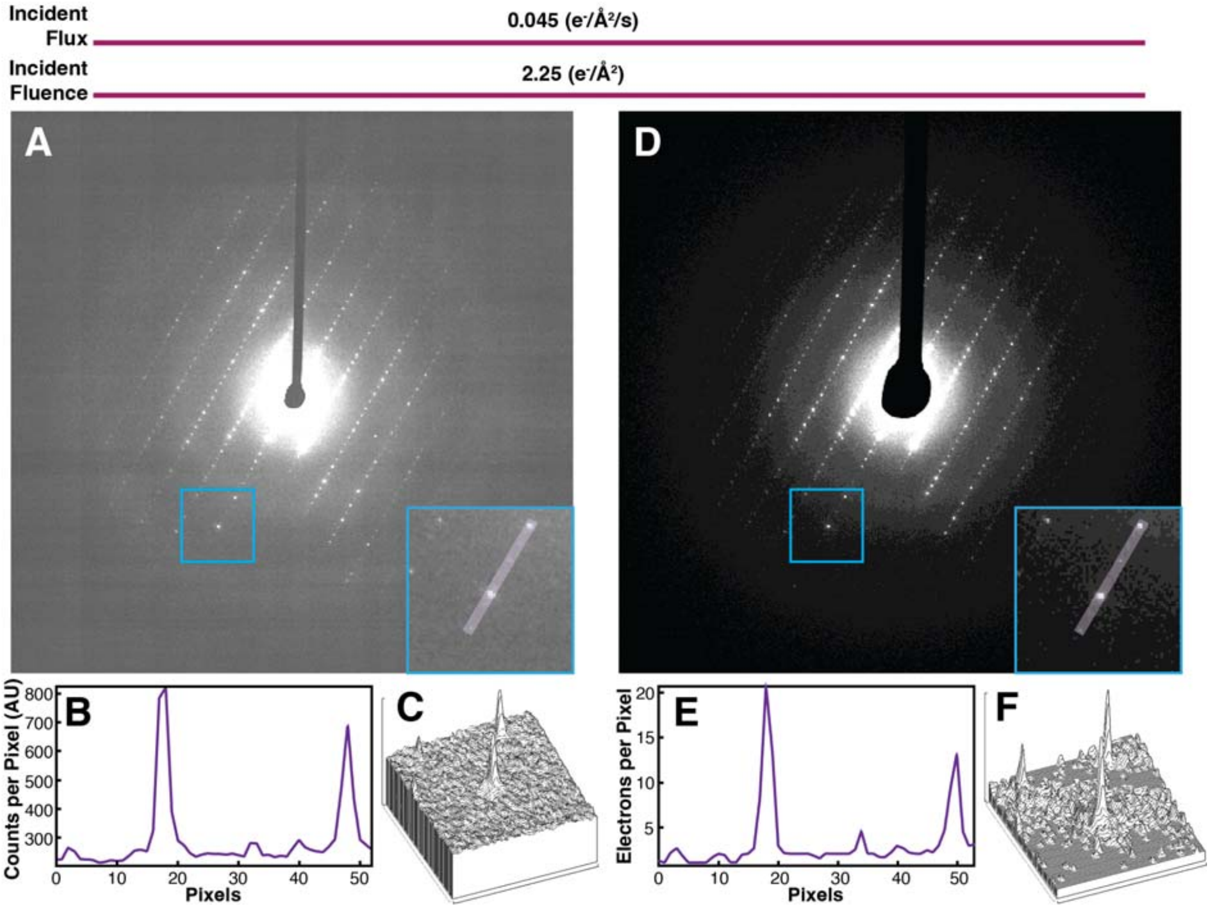

Supplementary figure 7. EBEC MicroED data collected from salen ligand crystals illuminated with an incident beam flux of 0.045 $e^-/Å^2/s$. Data from the same crystal was collected under identical conditions using a CETA-D (A) or Apollo (D) detector. Each pattern shows a maximum projection of measured counts over the same 2 degrees of each dataset. Insets show a region of the pattern containing visible reflections. An intensity profile (B,E) is calculated for the transparent line traversing the reflections in each inset. A 3D profile of each inset is also shown (C,F). The intensity range sampled in (A) ranges from 0 to 500 counts, while in (B) it ranges from 0 to 10 e^-.

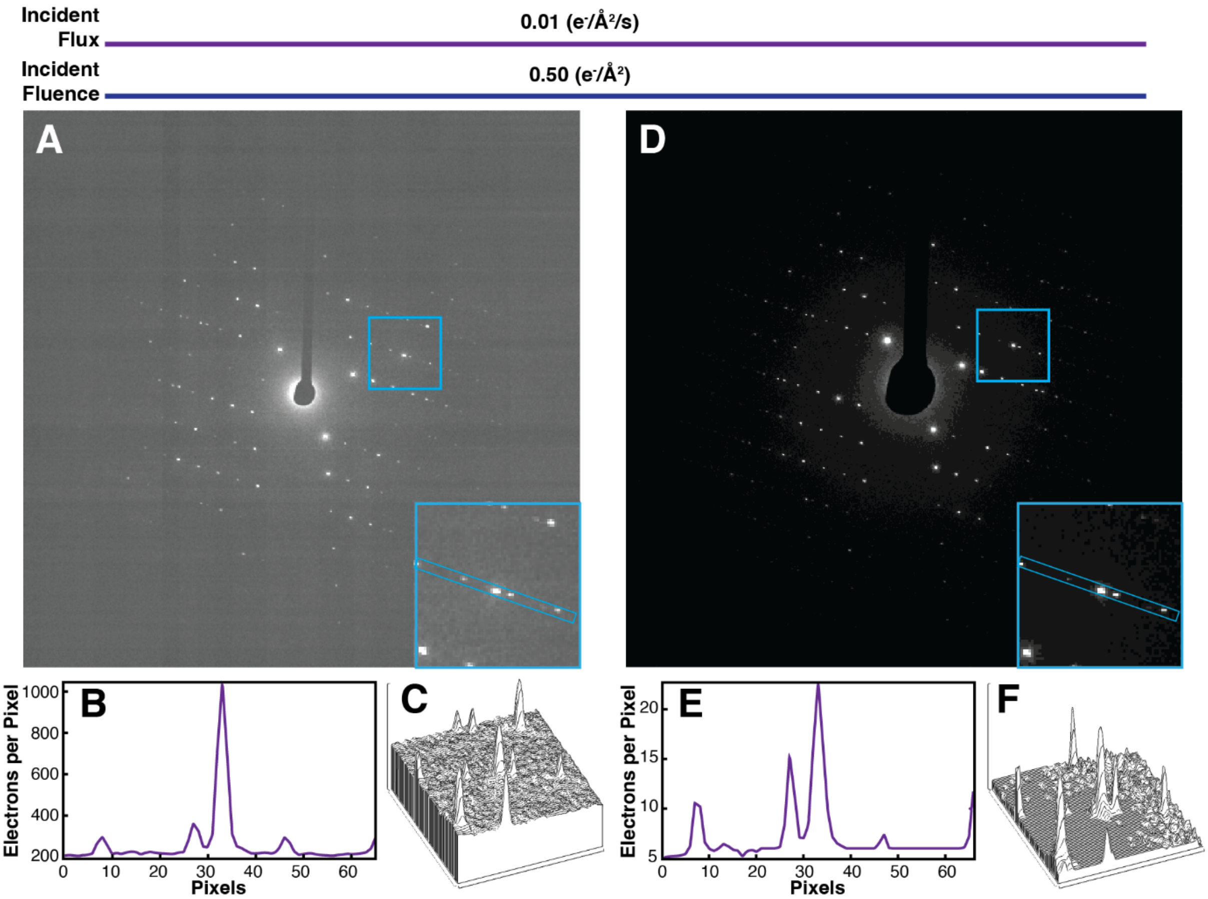

Supplementary figure 8. EBEC MicroED data collected from salen ligand crystals illuminated with an incident beam flux of 0.01 $e^{-}/Å^{2}/s$. Data from the same crystal was collected under identical conditions using a CETA-D (A) or Apollo (D) detector. Each pattern shows a maximum projection of measured counts over the same 2 degrees of each dataset. Insets show a region of the pattern containing visible reflections. An intensity profile (B,E) is calculated for the transparent line traversing the reflections in each inset. A 3D profile of each inset is also shown (C,F). The intensity range sampled in (A) ranges from 0 to 500 counts, while in (B) it ranges from 0 to 10 e^{-}.

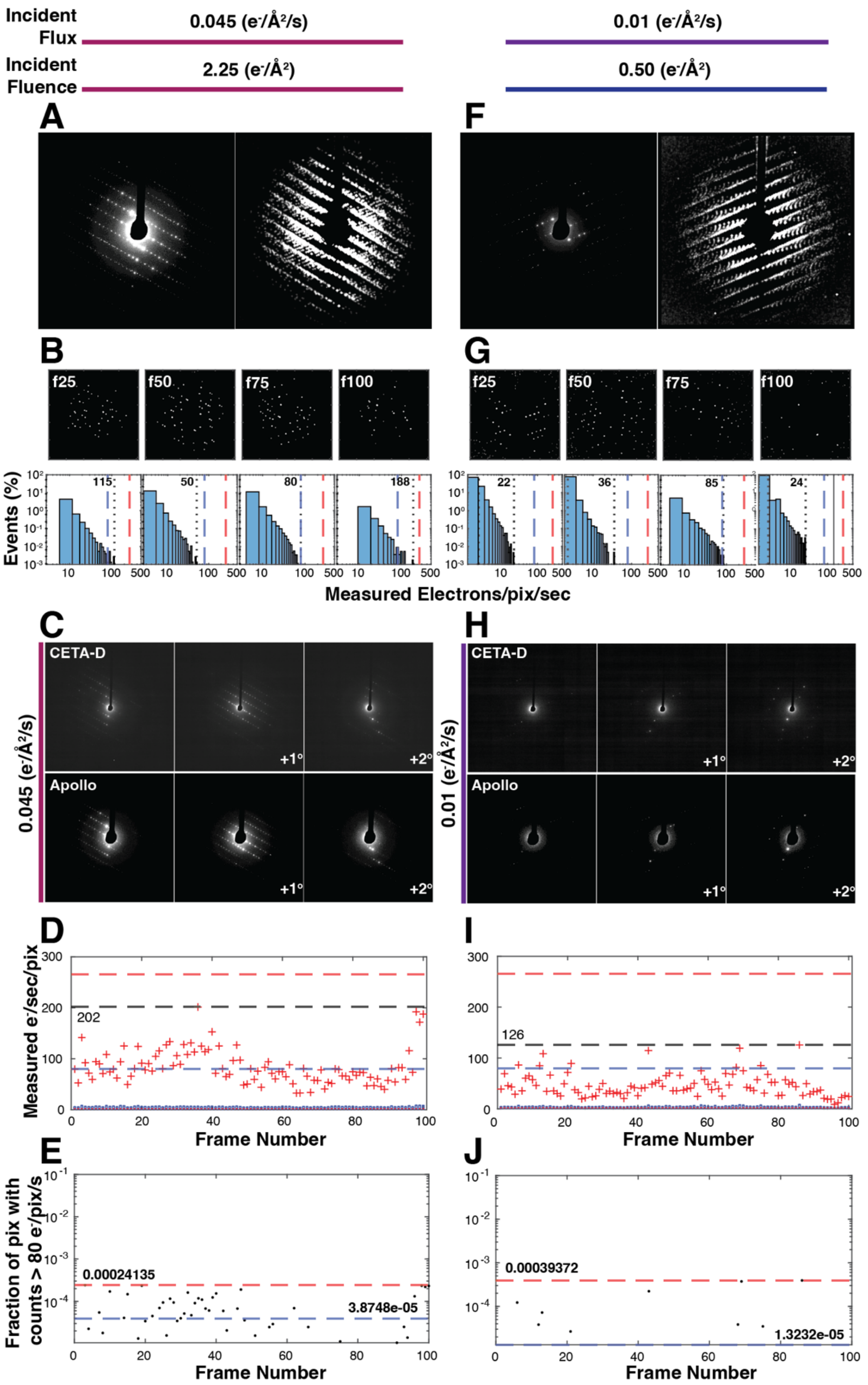

Supplementary figure 9. Comparison of diffraction patterns from salen ligand crystals illuminated by a high flux (A-E) or low flux (F-J) incident electron beam. Single patterns from a 100-frame MicroED dataset, spanning 100°, are shown alongside maximum projections of all identified

reflections across the measured wedge of data (A,F). Sets of reflections identified in patterns 25, 50, 75 and 100 of each dataset are shown above the respective histograms of counts for each pattern (B,G). In each histogram, dashed blue lines mark 80 e^-/pix/s, dashed red lines 266 e^-/pix/s, and dashed black lines the maximum counts in that frame. A series of three diffraction frames, 1° apart, shows pairs of patterns collected under identical conditions on the Apollo or CETA-D detectors (C,H). The maximum counts in each of the 100 images in the pattern are shown as red crosses in plots of the distribution of measured pixels per frame (D, I). Here, again, dashed blue lines mark 80 e^-/pix/s, dashed red lines 266 e^-/pix/s, and dashed black lines the maximum counts across all frames. The fraction of pixels in each frame with counts above 80 e^-/pix/s are noted in (E,J), where a dashed blue line shows the mean fraction of such pixels across all frames, while the dashed red line shows the maximum fraction of such pixels across all frames.

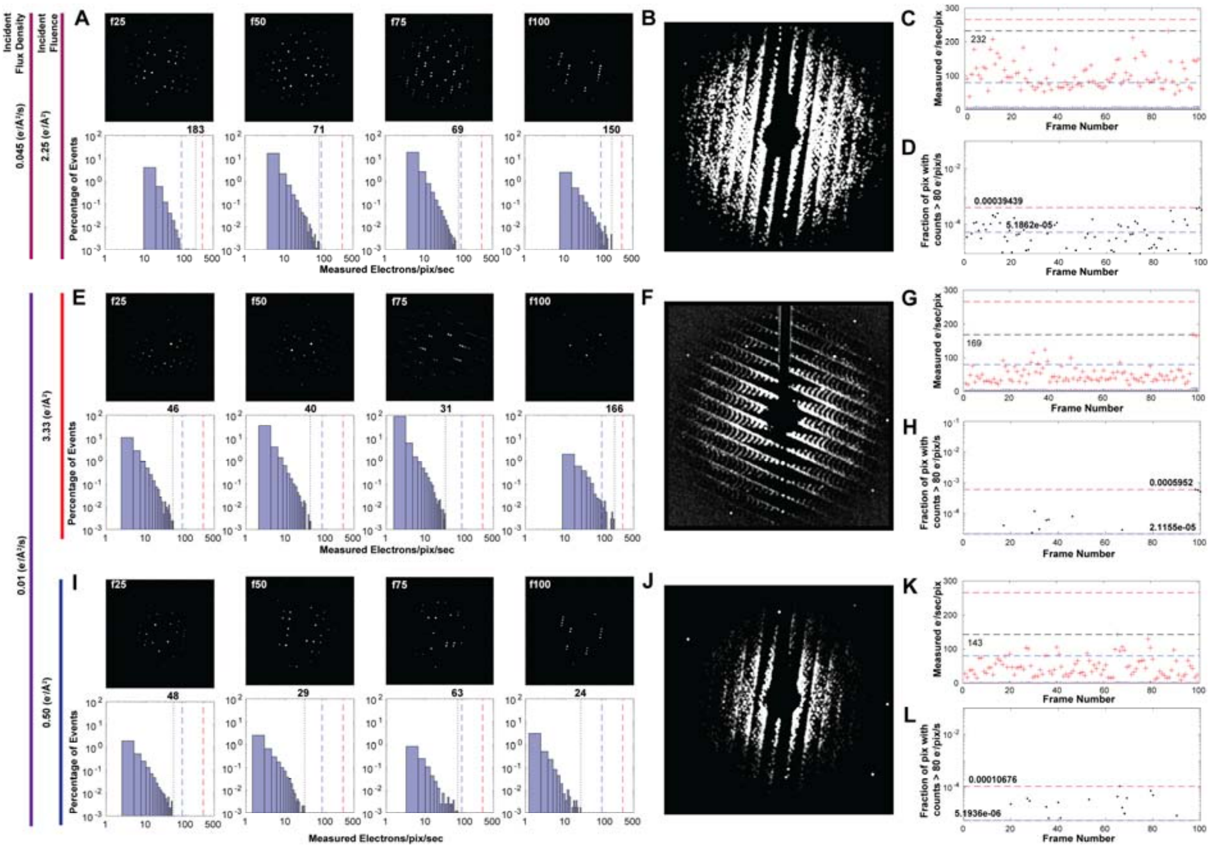

Supplementary figure 10. Representations of individual diffraction frames and histograms of electron count distributions for those frames, sampled from MicroED movies of salen ligand crystals. Reflections identified in each of four frames are shown; images correspond to frames 25, 50, 75 and 100 in a hundred-frame dataset. Three datasets sampled from three distinct salen ligand crystals are shown, with incident beam fluences of 2.25 (A-D) and 0.5 (E-H), and 3.33 (I-L) e^-/Å^2/s. On histogram plots, blue dashed lines denote 80 counted e^-/pix/s, red dashed lines indicate 266 e^-/pix/s, and black dashed lines indicate the maximum number of e^-/pix/s detected in the sampled frame. A dashed line and the number above it in histograms mark the maximum counts for each frame, in e^-/pix/s (A,E,I). In (B,F,J), binary images calculated from the summed diffraction pattern of the entire tilt series, displaying where reflections were detected in each movie. In (C,G,K), blue bars span the mean value of counts per frame +/- one standard deviation. A dashed blue line in each left graph indicates a count of 80 e^-/pix/s, a dashed red line shows the maximum anticipated count rate of 266 e^-/pix/s, and a dashed black line the maximum counts observed across all frames. Red crosses indicate the maximum number of counts observed in each of the 100 patterns of a movie. Each point in (D,H,L) indicates the fraction of pixels in a frame whose counts exceed 80 e^-/pix/s; points are absent where no pixels exceed that threshold. In (D,H,L), dashed blue lines indicate the average fraction for that set of points, and dashed red lines indicate the maximum fraction observed in the set; their numerical values are indicated.

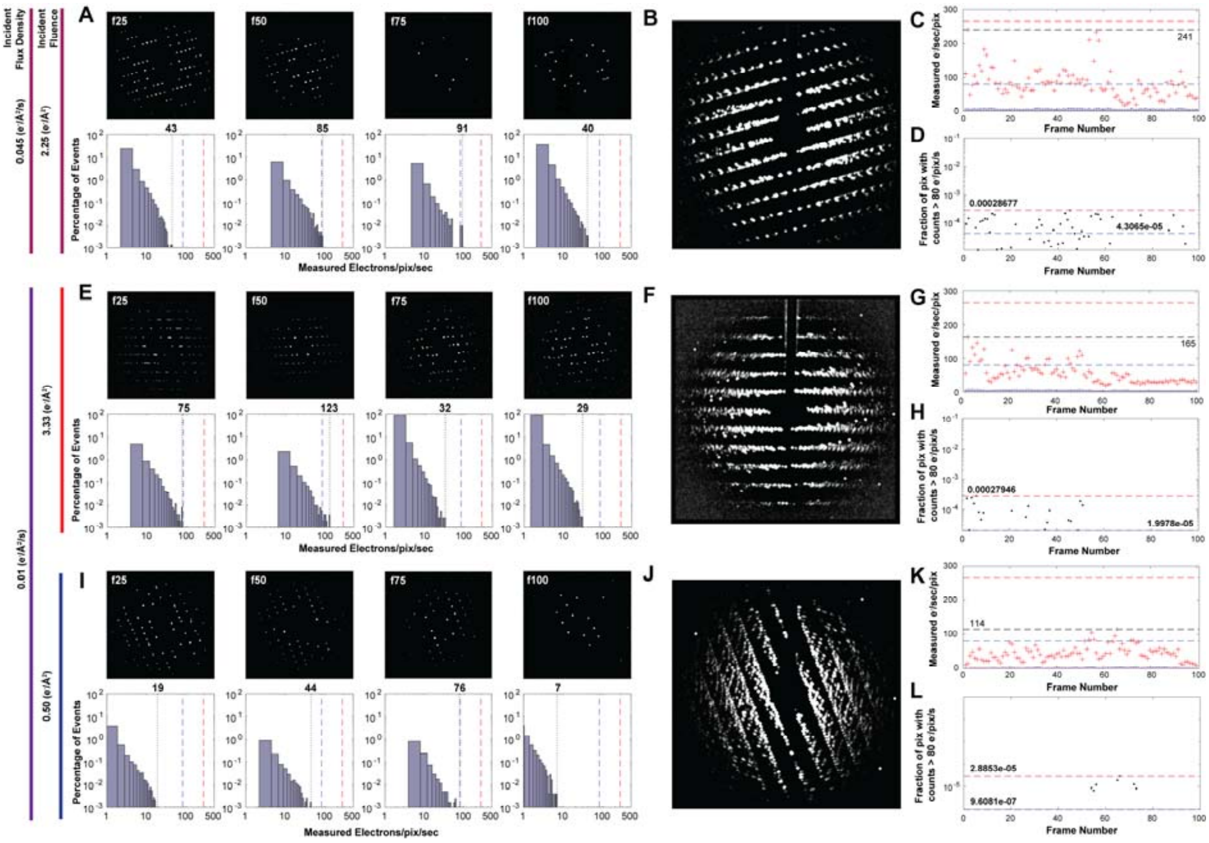

Supplementary figure 11. Representations of individual diffraction frames and histograms of electron count distributions for those frames, sampled from MicroED movies of biotin crystals. Reflections identified in each of four frames are shown; images correspond to frames 25, 50, 75 and 100 in a hundred-frame dataset. Three datasets sampled from three distinct salen ligand crystals are shown, with incident beam fluences of 2.25 (A-D) and 0.5 (E-H), and 3.33 (I-L) $e^-/Å^2/s$. On histogram plots, blue dashed lines denote 80 counted e^-/pix/s, red dashed lines indicate 266 e^-/pix/s, and black dashed lines indicate the maximum number of e^-/pix/s detected in the sampled frame. A dashed line and the number above it in histograms mark the maximum counts for each frame, in e^-/pix/s (A,E,I). In (B,F,J), binary images calculated from the summed diffraction pattern of the entire tilt series, displaying where reflections were detected in each movie. In (C,G,K), blue bars span the mean value of counts per frame +/- one standard deviation. A dashed blue line in each left graph indicates a count of 80 e^-/pix/s, a dashed red line shows the maximum anticipated count rate of 266 e^-/pix/s, and a dashed black line the maximum counts observed across all frames. Red crosses indicate the maximum number of counts observed in each of the 100 patterns of a movie. Each point in (D,H,L) indicates the fraction of pixels in a frame whose counts exceed 80 e^-/pix/s; points are absent where no pixels exceed that threshold. In (D,H,L), dashed blue lines indicate the average fraction for that set of points, and dashed red lines indicate the maximum fraction observed in the set; their numerical values are indicated.

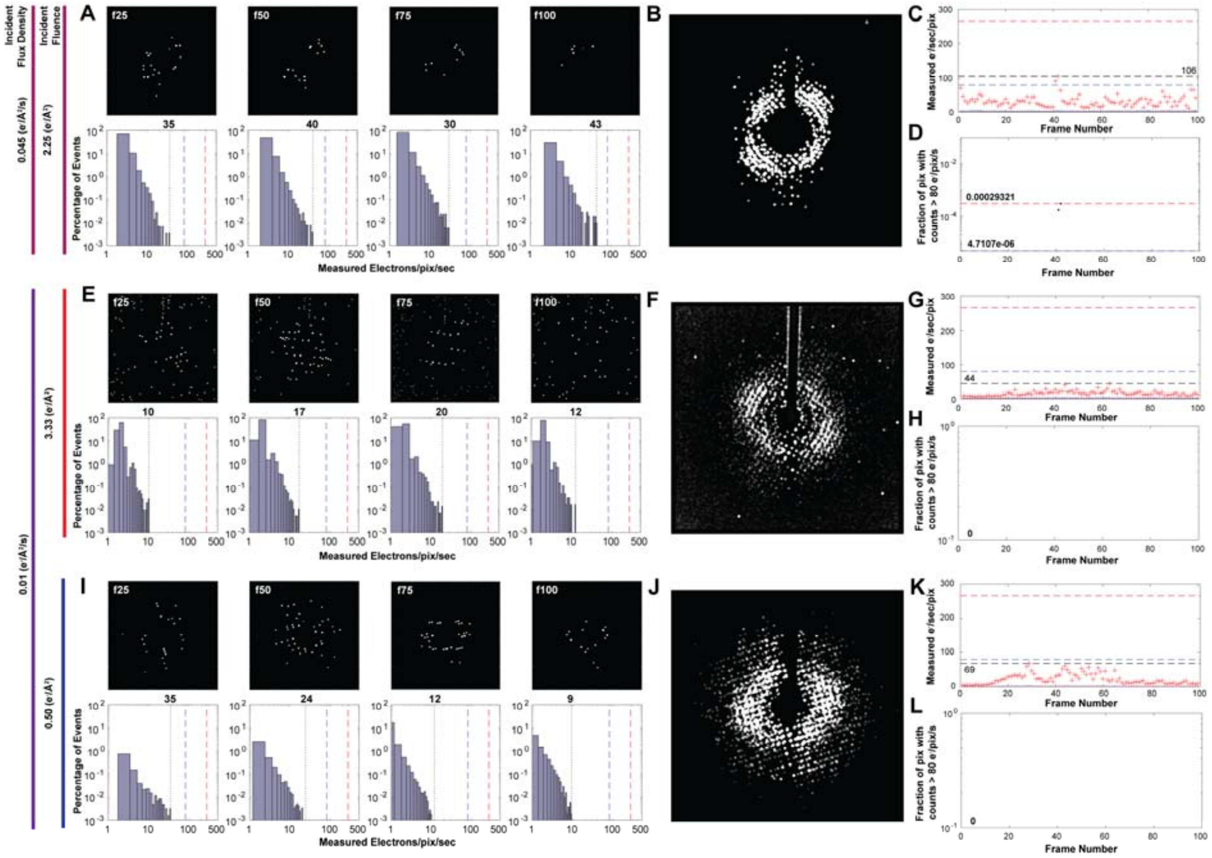

Supplementary figure 12. Representations of individual diffraction frames and histograms of electron count distributions for those frames, sampled from MicroED movies of thiostrepton crystals. Reflections identified in each of four frames are shown; images correspond to frames 25, 50, 75 and 100 in a hundred-frame dataset. Three datasets sampled from three distinct salen ligand crystals are shown, with incident beam fluences of 2.25 (A-D) and 0.5 (E-H), and 3.33 (I-L) $e^-/Å^2/s$. On histogram plots, blue dashed lines denote 80 counted $e^-/pix/s$, red dashed lines indicate 266 $e^-/pix/s$, and black dashed lines indicate the maximum number of $e^-/pix/s$ detected in the sampled frame. A dashed line and the number above it in histograms mark the maximum counts for each frame, in $e^-/pix/s$ (A,E,I). In (B,F,J), binary images calculated from the summed diffraction pattern of the entire tilt series, displaying where reflections were detected in each movie. In (C,G,K), blue bars span the mean value of counts per frame +/- one standard deviation. A dashed blue line in each left graph indicates a count of 80 $e^-/pix/s$, a dashed red line shows the maximum anticipated count rate of 266 $e^-/pix/s$, and a dashed black line the maximum counts observed across all frames. Red crosses indicate the maximum number of counts observed in each of the 100 patterns of a movie. Each point in (D,H,L) indicates the fraction of pixels in a frame whose counts exceed 80 $e^-/pix/s$; points are absent where no pixels exceed that threshold. In (D,H,L), dashed blue lines indicate the average fraction for that set of points, and dashed red lines indicate the maximum fraction observed in the set; their numerical values are indicated.

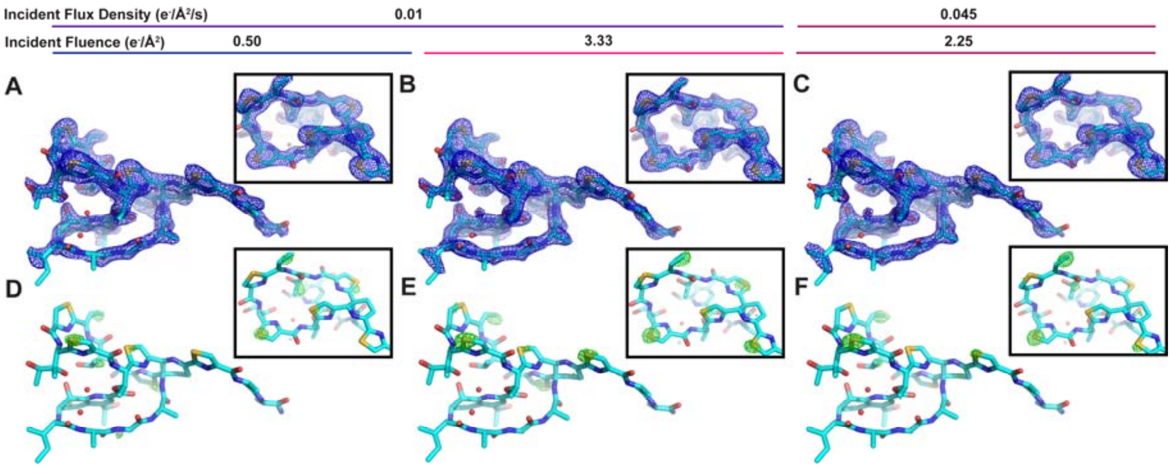

Supplementary figure 13. Structures of thiostrepton at 2.0 Å from EBEC MicroED data collected with different incident electron beam fluxes, where only the set of miller indices observed in all three experiments is was retained for refinement. Likewise, the same test set for calculation of R-free is used for all three trials. (A,B,C) display the results of rigid body refinement of data prepared in this way against the model of thiostrepton refined from 3.33 $e^{-}/Å^{2}$ at 1.5 Å resolution, where the $2F_o$-F_c map is displayed at 1.5σ levels. Beneath each is the same model superimposed with a green F_o-F_c map at 3σ, calculated from rigid body refinement of the measured data against a poly-alanine model of thiostrepton (D,E,F).

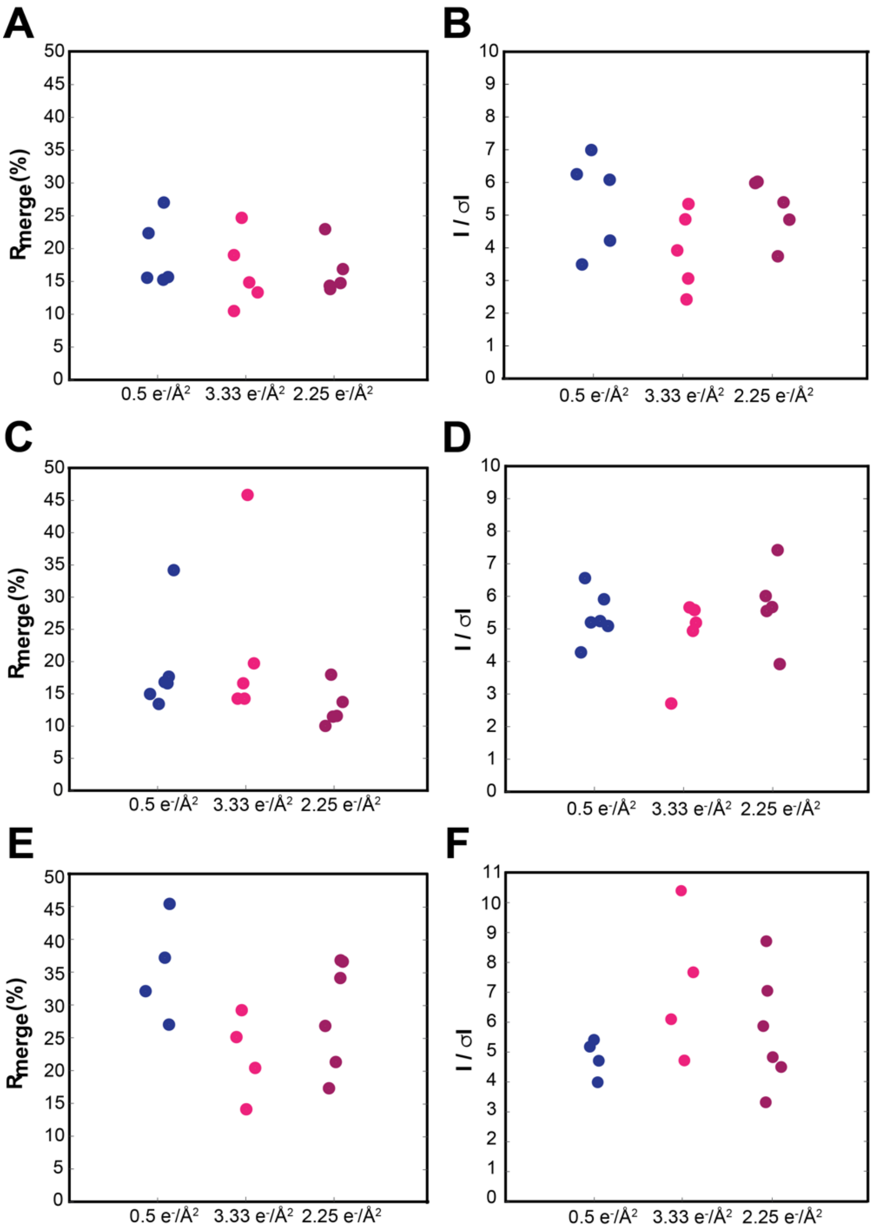

Supplementary figure 14. Analysis of EBEC MicroED data reduction statistics. Statistics for data collected with varying incident electron beam flux for salen ligand (A, B), biotin (C, D) and thiostrepton crystals (E, F). For each, the overall R_{merge} and I/sigma are shown for crystals sampled delivering a total fluence of 0.5 e$^-$/Å^2 (2 deg/s rotation, 2 integrated frames/s, 0.01 e$^-$/Å^2/s flux density, blue points), 3.33 0.5 e$^-$/Å^2 (0.3 deg/s rotation, 0.3 integrated frames/s, 0.01 e$^-$/Å^2/s flux density, pink points), and 2.25 e$^-$/Å^2 (2 deg/s rotation, 2 integrated frames/s, 0.045 e$^-$/Å^2/s flux density, magenta points). Reflections to a maximum resolution of 0.8 Å are considered for the salen ligand and biotin, and out to 2.0 Å for thiostrepton.

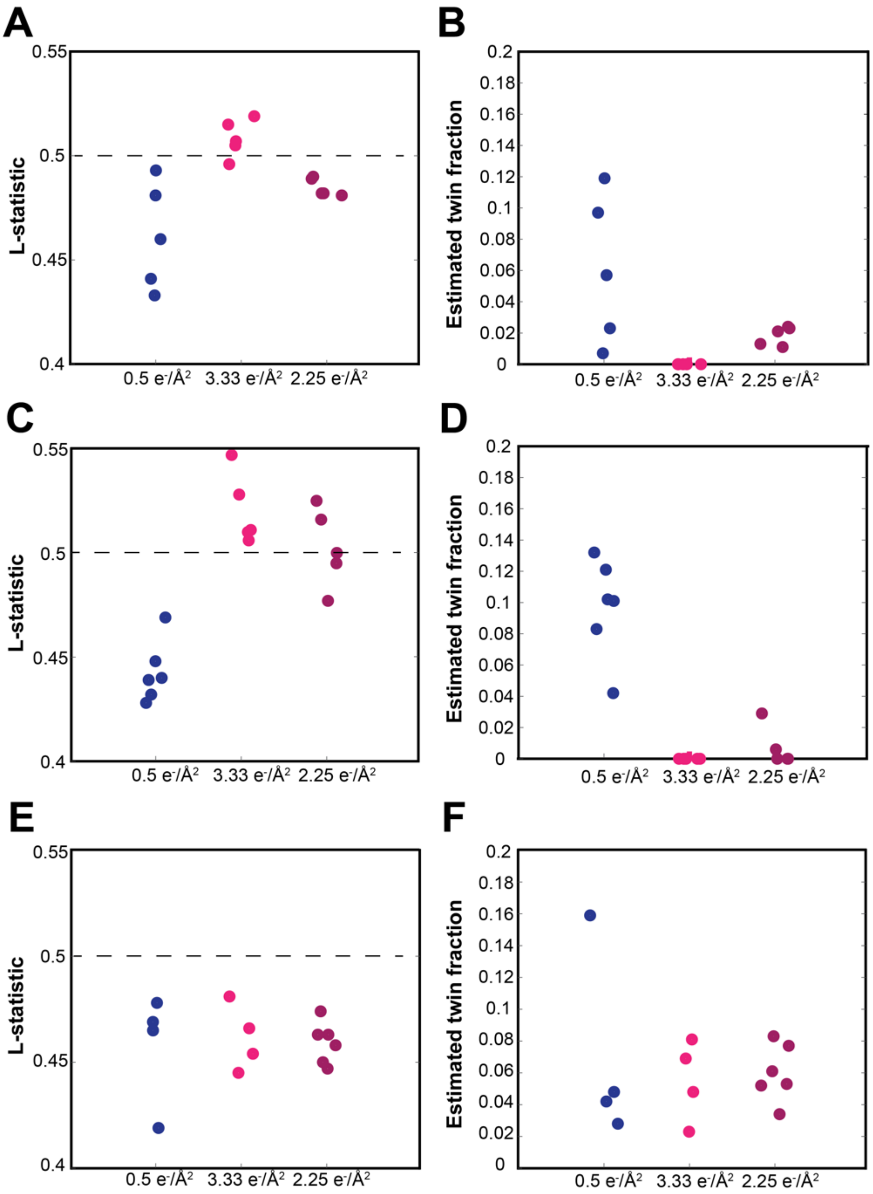

Supplementary figure 15. Analysis of L-test statistics vs. incident electron beam flux for datasets in Supplementary Figure 14. Plots show the L-statistic computed for each of the datasets of salen ligand (A), biotin (C) and thiostrepton (E) crystals, sampled delivering a total fluence of 0.5 $e^{-}/Å^{2}$ (2 deg/s rotation, 2 integrated frames/s, 0.01 $e^{-}/Å^{2}$/s flux density, blue points), 3.33 0.5 $e^{-}/Å^{2}$ (0.3 deg/s rotation, 0.3 integrated frames/s, 0.01 $e^{-}/Å^{2}$/s flux density, pink points), and 2.25 $e^{-}/Å^{2}$ (2 deg/s rotation, 2 integrated frames/s, 0.045 $e^{-}/Å^{2}$/s flux density, magenta points). A dashed black line indicates values above which data is considered perfectly un-twinned. Corresponding plots of the estimated twin fraction for each crystal are computed from the L statistic for salen ligand (B), biotin (D) and thiostrepton (F) crystals.

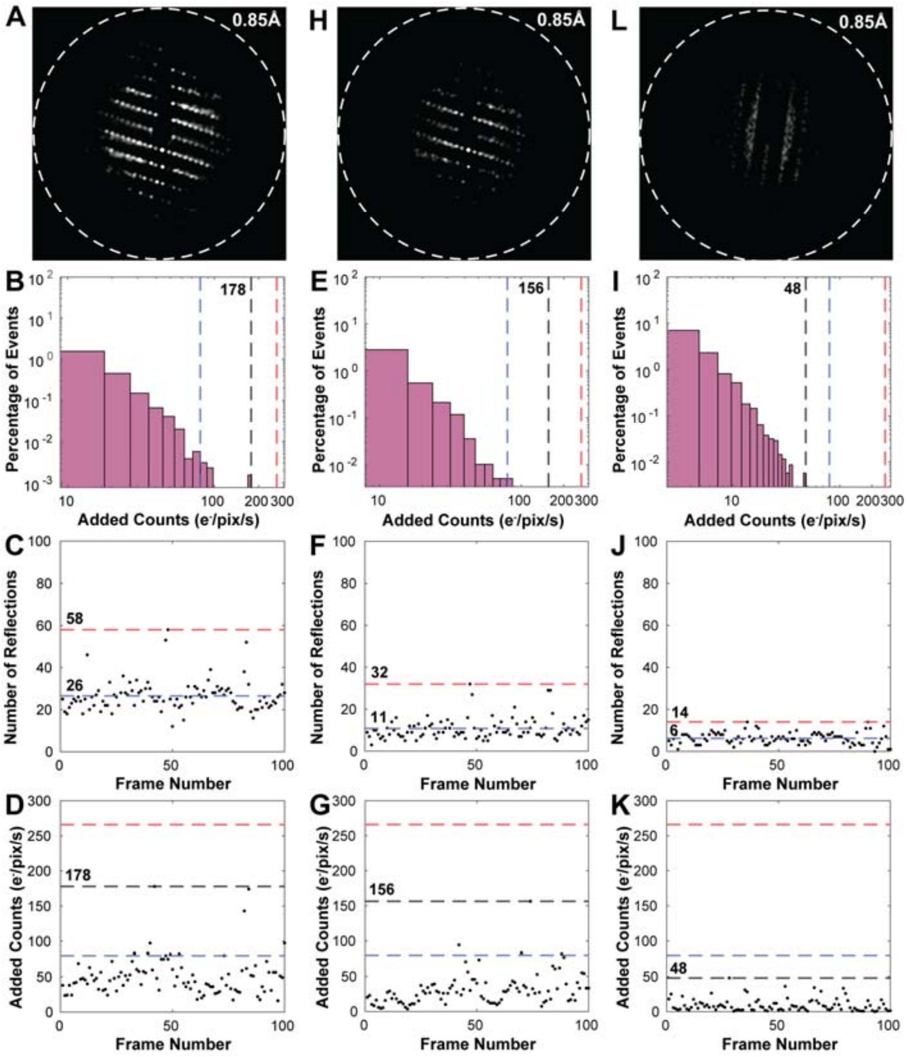

Supplementary figure 16. The impact of CL-adjustment on EBEC MicroED data collected from salen ligand crystals. Analyses of three representative crystal datasets are shown, across various incident beam flux and fluence settings: a flux of 0.045 $e^-/Å^2/s$ (A-D), 0.01 $e^-/Å^2/s$ (H-K); a fluence of 2.25 $e^-/Å^2$ (A-D), 0.5 $e^-/Å^2$ (H-G), 3.33 $e^-/Å^2$ (L-K). For each, a pattern shows the locations of all reflections that received a CL-adjustment (A,H,L), a histogram of all added counts across the entire movie (B,E,I), the total number of reflections adjusted per frame, across all frames in a dataset (C,F,J), and the maximum number of electron counts added for any given pixel in a frame, for all frames in a dataset (D,G,K). In (B,D,E,G,I,K), dashed red lines indicate 266 $e^-/pix/s$, dashed blue lines indicate 80 $e^-/pix/s$; dashed black lines indicate maximum electron counts added for the whole dataset. In (C,F,J), dashed red lines indicate maximum number of reflections adjusted in a given frame of the dataset; dashed blue lines indicate the mean number of reflections adjusted per frame over the dataset. Resolution rings are labeled in (A,H,L).

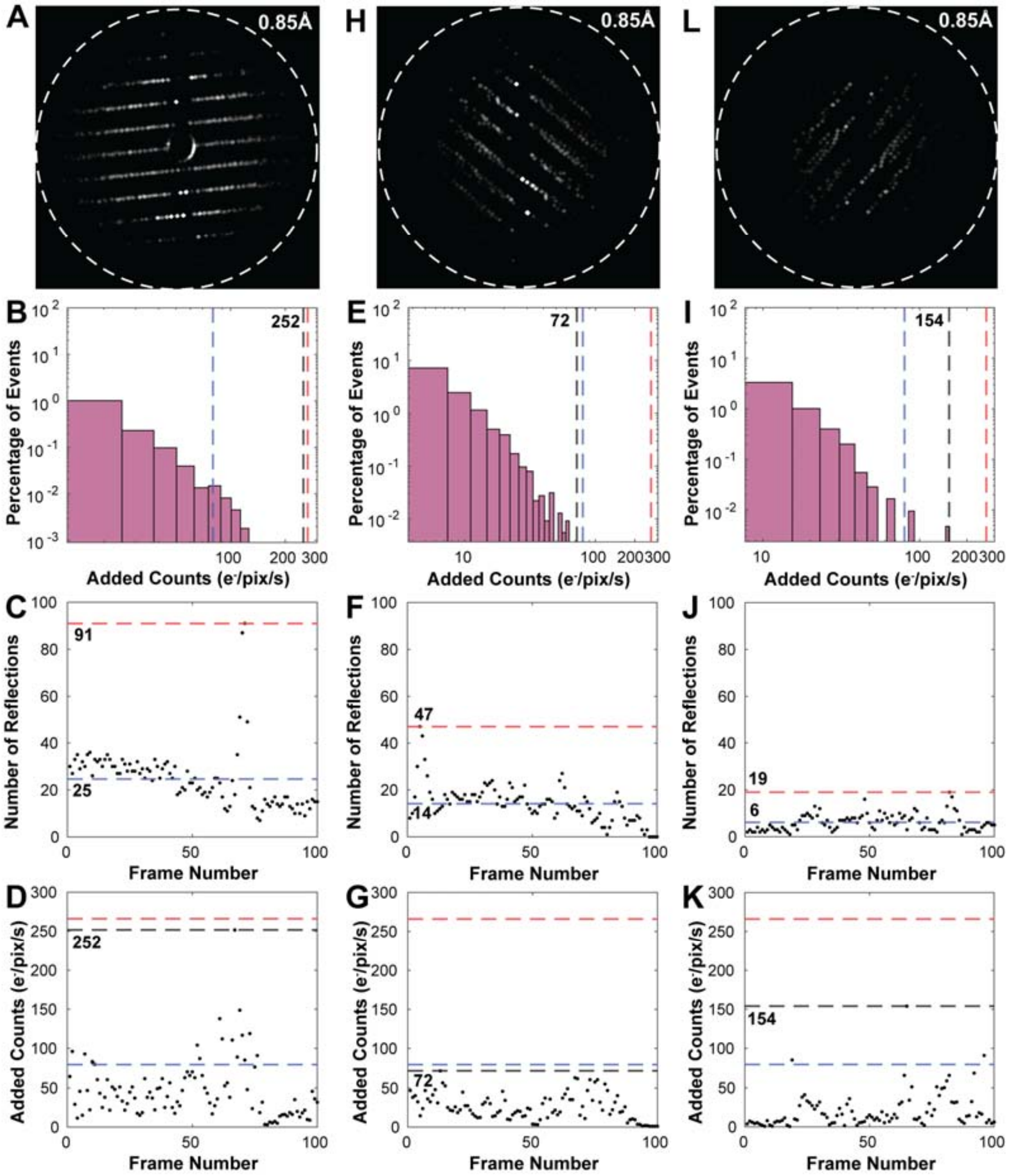

Supplementary figure 17. The impact of CL-adjustment on EBEC MicroED data collected from biotin crystals. Analyses of three representative crystal datasets are shown, across various incident beam flux and fluence settings: a flux of 0.045 e^-/Å^2/s (A-D), 0.01 e^-/Å^2/s (H-K); a fluence of 2.25 e^-/Å^2 (A-D), 0.5 e^-/Å^2 (H-G), 3.33 e^-/Å^2 (L-K). For each, a pattern shows the locations of all reflections that received a CL-adjustment (A,H,L), a histogram of all added counts across the entire movie (B,E,I), the total number of reflections adjusted per frame, across all frames in a dataset (C,F,J), and the maximum number of electron counts added for any given pixel in a frame, for all frames in a dataset (D,G,K). In (B,D,E,G,I,K), dashed red lines indicate 266 e^-/pix/s, dashed blue lines indicate 80 e^-/pix/s; dashed black lines indicate maximum electron counts added for the whole dataset. In (C,F,J), dashed red lines indicate maximum number of reflections adjusted in a given frame of the dataset; dashed blue lines indicate the mean number of reflections adjusted per frame over the dataset. Resolution rings are labeled in (A,H,L).

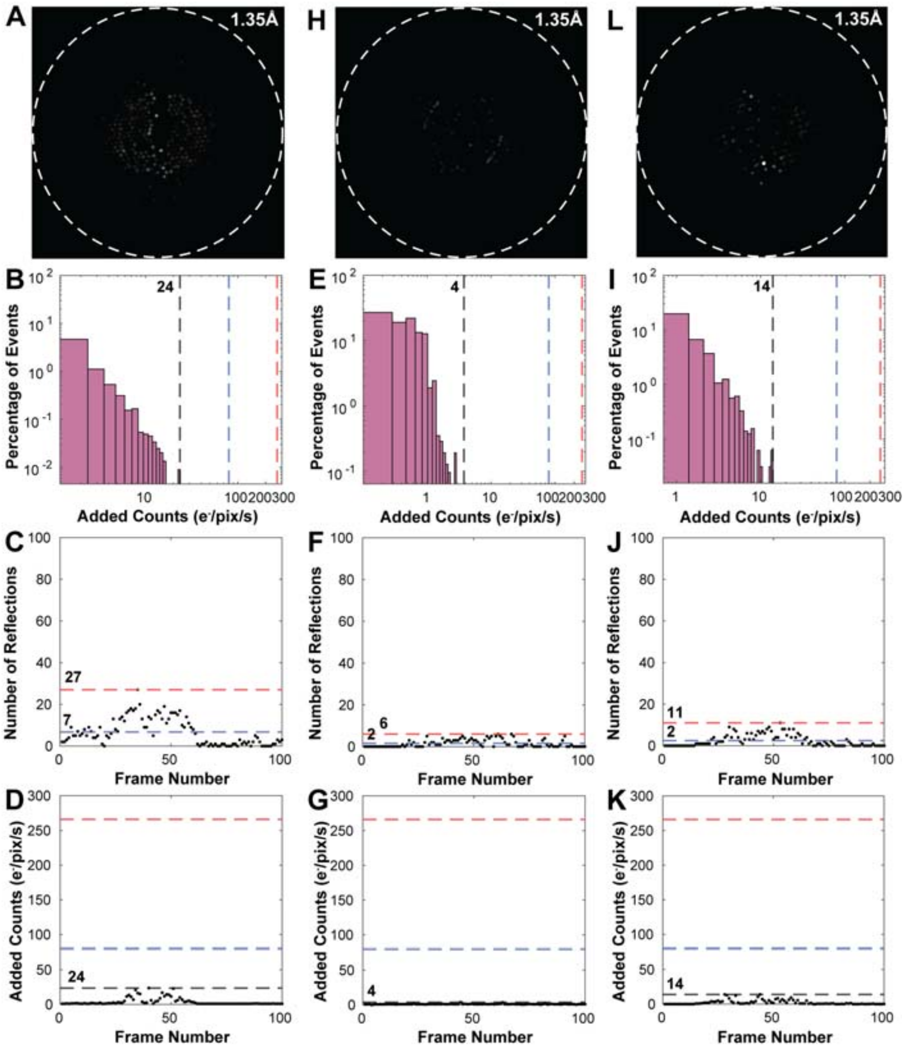

Supplementary figure 18. The impact of CL-adjustment on EBEC MicroED data collected from thiostrepton crystals. Analyses of three representative crystal datasets are shown, across various incident beam flux and fluence settings: a flux of 0.045 $e^-/Å^2/s$ (A-D), 0.01 $e^-/Å^2/s$ (H-K); a fluence of 2.25 $e^-/Å^2$ (A-D), 0.5 $e^-/Å^2$ (H-G), 3.33 $e^-/Å^2$ (L-K). For each, a pattern shows the locations of all reflections that received a CL-adjustment (A,H,L), a histogram of all added counts across the entire movie (B,E,I), the total number of reflections adjusted per frame, across all frames in a dataset (C,F,J), and the maximum number of electron counts added for any given pixel in a frame, for all frames in a dataset (D,G,K). In (B,D,E,G,I,K), dashed red lines indicate 266 e^-/pix/s, dashed blue lines indicate 80 e^-/pix/s; dashed black lines indicate maximum electron counts added for the whole dataset. In (C,F,J), dashed red lines indicate maximum number of reflections adjusted in a given frame of the dataset; dashed blue lines indicate the mean number of reflections adjusted per frame over the dataset. Resolution rings are labeled in (A,H,L).

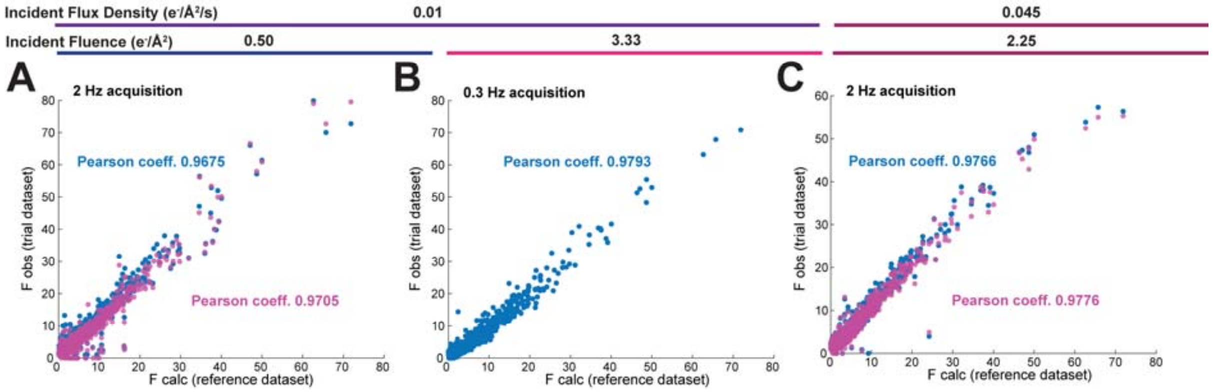

Supplementary figure 19. Plots of observed structure factors for the three representative EBEC datasets from salen ligand crystals used for structure determination in Table 1 and Figure 3, against calculated structure factors from a high quality MicroED structure of the salen ligand determined by conventional methods at 200 keV. For data collected at incident flux density of 0.01 $e^-/Å^2/s$ (A-B), 0.045 $e^-/Å^2/s$ (C), with total fluence of 0.5 $e^-/Å^2$ (A), 3.33 $e^-/Å^2$ (B), and 2.25 $e^-/Å^2$ (C), structure factors for observed reflections are plotted against calculated structure factors for the same indices in blue. Additionally, for data acquired at 2 Hz, observed structure factors following application of the CL adjustment are plotted in magenta.
